# Mobilization of CD11b^+^/Ly6c^hi^ monocytes causes multi organ dysfunction syndrome in acute pancreatitis

**DOI:** 10.3389/fimmu.2022.991295

**Published:** 2022-10-10

**Authors:** Anika Wilden, Juliane Glaubitz, Oliver Otto, Doreen Biedenweg, Matthias Nauck, Matthias Mack, Silvia Ribback, Barbara M. Bröker, Sabrina Freiin von Rheinbaben, Markus M. Lerch, Ali Alexander Aghdassi, Frank Ulrich Weiss, Matthias Sendler

**Affiliations:** ^1^ Department of Medicine A, University Medicine Greifswald, Greifswald, Germany; ^2^ Center for Innovation Competence: Humoral Immune Reactions in Cardiovascular Disorders, University of Greifswald, Greifswald, Germany; ^3^ Department of Medicine B, University Medicine Greifswald, Greifswald, Germany; ^4^ Institute of Clinical Chemistry and Laboratory Diagnostics, University Medicine Greifswald, Greifswald, Germany; ^5^ Deutsches Zentrum für Herz-Kreislauf-Forschung (DZHK) (German Centre for Cardiovascular Research), Partner Site Greifswald, University Medicine, Greifswald, Germany; ^6^ Department of Internal Medicine II, University Hospital Regensburg, Regensburg, Germany; ^7^ Institute of Pathology, Universitat Greifswald, Greifswald, Mecklenburg-Vorpommern, Germany; ^8^ Department of Immunology, University Medicine Greifswald, Greifswald, Germany

**Keywords:** monocytes, MODS, acute pancreatitis, CCR2, MCP-1

## Abstract

**Objective:**

Acute pancreatitis (AP) is an inflammatory disorder, the severe form of which is burdened with multi-organ dysfunction and high mortality. The pathogenesis of life –threatening organ complications, such as respiratory and renal failure, is unknown.

**Design:**

Organ dysfunction was investigated in a mouse model of AP. The influence of monocytes and neutrophils on multi organ dysfunction syndrome (MODS) was investigated *in vivo* by antibody depletion. Using real-time-fluorescence and deformability-cytometry (RT-DC) analysis we determined the mechanical properties of neutrophils and monocytes during AP. Furthermore, blood samples of pancreatitis patients were used to characterize severity-dependent chemokine profiles according to the revised Atlanta classification.

**Results:**

Similar to AP in humans, severe disease in the mouse model associates with organ dysfunction mainly of lung and kidney, which is triggered by a mobilisation of Ly6g^-^/CD11b^+^/Ly6c ^hi^ monocytes, but not of Ly6g^+^/CD11b^+^ neutrophils. Monocyte depletion by anti-CCR2 antibody treatment ameliorated lung function (oxygen consumption) without interfering with the systemic immune response. RT-DC analysis of circulation monocytes showed a significant increase in cell size during SAP, but without a compensatory increase in elasticity. Patient chemokine profiles show a correlation of AP severity with monocyte attracting chemokines like MCP-1 or MIG and with leukocyte mobilisation.

**Conclusion:**

In AP, the physical properties of mobilized monocytes, especially their large size, result in an obstruction of the fine capillary systems of the lung and of the kidney glomeruli. A selective depletion of monocytes may represent a treatment strategy for pancreatitis as well as for other inflammation-related disorders.

## Introduction

Acute pancreatitis (AP) is one of the most common non-malignant gastroenterological disorders leading to hospitalisation of patients (1). In most cases the disease has a mild self-limiting course, but 20% of patients develop severe acute pancreatitis, which is associated with systemic complications. Infected necrosis and/or persistent organ failure are the most perilous complications leading to significant morbidity and mortality (1, 2). AP can rapidly progress towards moderately severe or severe AP (SAP), significantly increasing the length of in-hospital stays and ultimately leading to significant healthcare expenditures (3). Until today there is no established causative treatment for AP or SAP.

According to the revised Atlanta classification, the severe form of acute pancreatitis is defined by the occurrence of persistent organ failure or the presence of a systemic inflammatory response syndrome (SIRS) for more than 48h (4). Pancreatitis-induced SIRS is the suggested trigger mechanism for organ damage, affecting mainly the pulmonary, the renal or the cardiovascular system (2). While SIRS is held responsible for early organ damage like acute respiratory distress syndrome (ARDS) or acute kidney injury (AKI) (2, 5), the compensatory anti-inflammatory response syndrome (CARS) is believed to be responsible for the infection of pancreatic necrosis and late organ failure. The release of pro-inflammatory cytokines and chemokines can induce severe systemic inflammation, resulting in multi-organ dysfunction syndrome (MODS) (5). Pancreatitis-related organ complications frequently include the respiratory system (2, 6). IL-6 trans-signalling (7) or MCP-1-mediated monocyte/granulocyte mobilisation (8) are associated with lung damage during SAP. However, the precise pathomechanism behind pancreatitis-induced ARDS is not well understood, one reason being the lack of reproducible SAP animal models

Our aim for the present study was to investigate the cause of secondary organ damage/MODS in a mouse model of SAP induced by partial pancreatic duct ligation and hormonal hyperstimulation. In this model the mice develop the full spectrum of disease characteristics of SAP in humans (9, 10), including pancreatic necrosis and prominent damage of the respiratory and renal systems. After the induction of AP cells of the innate immune system, especially monocytes and neutrophils, are mobilized and migrate into the inflamed pancreatic tissue. We used specific antibody treatment to deplete Ly6g^+^ neutrophils or CCR2^+^ monocytes and analysed the local and systemic immune responses, and how this might impact on the development of organ complications. Our results indicate that during severe inflammatory reactions like pancreatitis or sepsis a general disease mechanism causes damage of secondary organs by microcapillary occlusion from enlarged monocytes, which offers a therapeutic option for a causative treatment of MODS.

## Material and methods

### Animal model

Wildtype C57Bl/6 mice were purchased from Charles River Laboratories (Sulzfeld, Germany). SAP was induced by partial duct ligation of the pancreatic duct and an additional caerulein injection at day 2 (50mg/kg bodyweight), as described previously (9–12), untreated mice were used as controls. Animals were sacrificed at day 3 after surgical duct ligation. To deplete monocytes and neutrophils mice were treated with anti-Ly6G antibody (13) (BioXcell, Lebanon USA, BP0075-1), with rat anti-mouse-CCR2 antibody MC-21 (14) (a kind gift from Matthias Mack, Regensburg Germany, lot 1480/02) and as control with isotype IgG2a (BioXcell, BP0089) or isotype IgG2b (BioXcell BE0090). Anti-Ly6G antibody and isotype IgG2a antibody were injected i.p. with 200µg in 200µl PBS one day before and the day after duct ligation. Anti-CCR2 antibody and Isotype IgG2b antibody were injected i.p. with 20µg in 200µl PBS at the day of duct ligation and one and two days after surgery. At minimum 6 animals were used per group. Mice were sacrificed 72 hours after duct ligation and organs were used for analysis. Serum was stored at -80°C. Pancreas, lung and kidney were fixed in 4.5% formaldehyde and embedded in paraffin, embedded in TissueTec or frozen in liquid nitrogen for rtPCR analysis. Spleen and lung were taken for FACS analysis.

Perfusion with fluorescein conjugated concanavalin a (con a, Vector laboratories Inc. USA, FL-1001). Animals were anaesthetized using ketanes/xylasin and the thorax was surgically opened. 200µl con a solution (10% in PBS) was slowly injected into the right ventricle over 1min. Immediately afterwards, the lungs were removed, and one part fixed in 4% paraformaldehyde solution while the other part was stored at -80°C for further processing. Paraformaldehyde fixed lung was embedded with TissueTec for cryo sections.

### Human sample collection

Acute pancreatitis serum samples were collected at the university medicine Greifswald between the years 2005 and 2020, after approval by the Ethical committee of the university medicine Greifswald (III UV 91/03). All patients gave written and informed consent.

### Antibodies and reagents

The following antibodies were used for FACS staining, IHC staining and immunofluorescence staining: Anti-mouse CD4 BV650 (BioLegend 100546), anti-mouse-CD11b PerCP Cy55 (BioLegend, 101228), anti-mouse-Ly6G BV421 (BioLegend, 127628), anti-mouse-Ly6C BV605 (BioLegend 128036), anti-CD25-PECy7 (BioLegend, 102016), anti-CD69 BV510 (BioLegend, 104532), anti-CD8a BV605 (BioLegend, 100743), anti-FoxP3 APC (Miltenyi Biotec, 130-111-601), anti-mouse-CD11b (abcam, Ab133357), anti-mouse-CD68 (antibody-online, ABIN181836), anti-CCR2 (abcam, ab273050) anti-Ki67 (Bethyl, IHC-00375), anti-mouse-CD11b (abcam, Ab133357), anti-mouse-Ly6g (abcam, Ab25377), anti-mouse-Cystatin C (Novus biologicals, NB100-1033), anti-VE-cadherin (abcam, ab7047-50) anti-rabbit-HRP (DAKO, K4003), anti-mouse-HRP (DAKO, K4001). Caerulein was obtained from Sigma Aldricht (C9026-1MG, Munich, Germany), human myeloperoxidase from Calbiochem (Cat# 475911). All antibodies used in this study had been tested and established in previous studies (11, 12, 15). The concentration of the antibodies was used according to the manufacturer’s instructions.

### Flow cytometry analysis of spleen

To analyse splenocytes, spleen was taken and mashed through a 70µm cell strainer. Cells were washed with PBS and centrifuged at 300g for 6 minutes. To lyse erythrocytes pellet was resuspended in 1 ml lysis buffer for 5 minutes. After washing with PBS and centrifugation at 300g for 6 minutes cells were stained with fluorescence antibodies. Extracellular staining was performed with surface antibodies (1:50): Anti-CD4, anti-CD11b, anti-Ly6G, anti-Ly6C, anti-CD25, anti-CD69, anti-CD8a. To block non-specific Fc mediated interactions cells were pre-treated with anti-mouse CD16/CD32 antibody for 5 min (1:50). Extracellular antibodies were incubated for 30 minutes at 4°C. Cells were washed with PBS and centrifuged at 300g for 6 minutes. Pellet was resuspended in 150µl FACS buffer and measured immediately. Our multicolour panels contain up to 8 different antibodies. To distinguish between specific and non-specific staining we used single stained controls. The single stained control determines the intensity of expression in a mixed antibody panel, using unmixed fluorochrome-labeled antibody with compensation beads (Anti-Rat and Anti-Hamster Ig κ/Negative Control Compensation Particles Set, 552845, BD). This method allows a compensation correction for spectral overlap (16). Data were analyzed with BD FACS DIVA Software and FlowJo.

### Flow cytometry analysis of lung

To analyze leukocytes from lung, lungs were removed from mice and were dissociated according to the protocol of lung dissociation kit from Miltenyi (130-095-927, Bergisch Gladbach, germany). Staining for flow cytometry was performed as described in splenocyte staining. Data were analyzed with BD FACS DIVA Software and FlowJo.

### Flow cytometry analysis of blood

Fresh blood was collected in in BD Vacutainer K2E EDTA tubes (367864, BD) and 100µL blood was stained as described in splenocytes staining.

### Serum amylase and lipase

Serum amylase and lipase measurement was performed with colorimetric assay from Roche/Hitacho (Amyl Ref 11876473316; Lip Ref 11821792216).

### Oxygen saturation of blood

Arterial Blood was taken with a capillary from arteria carotis communis and was measured with BGA analysis machine (ABL90 FLEX, Radiometer GmbH, Germany). Carotis communis was prepared as described previously. Parameters like pO_2_, pCO_2_, glucose, lactat, etc. were measured and analysed.

### MPO activity

MPO measurement was performed like previously described (11, 13, 17, 18). Lung tissue was homogenized on ice in 20mM potassium phosphate buffer (pH 7.4) and centrifuged at 14.000 rcf. The pellet was resuspended in potassium phosphate buffer (pH 6.0) containing 0.5% cetyltrimethylammoniumbromide. Suspension was frozen and thawed in 4 cycles and centrifuged at 14.000 rcf. MPO activity was measured in 50 mM potassium phosphate buffer (pH 6.0) containing 0.53 mM O-dianisidine and 0.15 mM H_2_O_2_. The MPO activity was measured as kinetic over time with a Spectramax Spectrophotometer (Molecular devices). Purified human myeloperoxidase was used as standard, the final MPO activity was calculated against protein content of the samples.

### Histology, immunohistochemistry and immunofluorescence staining

For H&E staining the lung, pancreas and kidney were embedded in paraffin, 3µm thick slides were cut by microtome. Slides were deparaffined in Xylol, and hydrated in methanol followed by decreased ethanol concentrations, hematoxylin and eosin staining was performed as described previously (10, 13). For lung analysis slides were scanned with slide scanner (Pannoramic MDI BF/FL; Sysmex, Norderstedt, Germany) and analysis were made with Software Pattern Quant from Sysmex Quant Center.

For immunohistochemistry staining slides were dewaxed and heated in antigen retrieval solution (target retrieval solution, ref. S1699, Dako Agilent, Santa Clara, USA) for 30min under pressure using a steam pressure cooker. Rabbit anti-mouse-CD11b antibody (abcam, cambride UK, Ab133357) was used in a concentration of 1:400 overnight at 4°C. Anti-rabbit antibody was incubated for 1 hour at RT. DAB Kit (Vector, Burlingame USA, SK4100) was used to visualize CD11b positive cells.

For immunofluorescence staining TissueTec embedded slides were used. Rabbit-anti-mouse-CD11b, Ly6g, CD68, CCR2 or VE-cadherin antibody was used as 1:400. Secondary antibody was used in a concentration of 1:200. The following secondary antibodies were used: anti-rabbit-Cy3 (111-165-144, Jackson ImmunoResearch), anti-rat-Cy3 (112-165-062, Jackson ImmunoResearch), anti-mouse-FITC (115-095-146, Jackson ImmunoResearch), anti-rat-Cy5 (112-175-143, Jackson ImmunoResearch). DAPI was used for nucleus staining. All slides were scanned with Pannoramic slide scanner and analysis were made with Sysmex Quant Center Nucleus or Pattern.

### Cytokines in serum

The chemokines MCP-1, MIG, IP-10, IL8 and complement system anaphylatoxins C3a, C4a and C5a were measured in human serum from patients with severe, moderate and mild form of acute pancreatitis. Measurements were performed with CBA Kits from BD (Human Anaphylatoxin Kit 561418BD; Human Chemokine Kit 552990; BD San Jose, USA). Murine chemokines and cytokines were measured by BD cytometric bead array (CBA) mouse inflammation kit in serum of mice.

### Real time fluorescence and deformability cytometry

Mechanical characterization of monocytes was performed in whole blood and lung monocytes isolated from pancreatic duct-ligated mice as well as untreated controls, respectively. For monocyte identification in whole blood, cells were labeled with Ly6g (APC conjugated) and CD11b (FITC conjugated) fluorescent antibodies. Lung monocytes were isolated (lung dissociation Kit, Miltenyi) by usage of monocytes isolation kit (EasySep mouse Monocyte Isolation Kit, Stemcell, 19861A). After isolation lung monocytes are resuspended in PBS (without Ca^2+^ and Mg^2+^) complemented with 0.6% (w/v) Methylcellulose to a concentration of approximately 10 x 10^6^ cells per ml.

High-throughput mechanical analysis was done by real-time fluorescence and deformability cytometry using the AcCellerator system with fluorescence extension (Zellmechanik Dresden GmbH) (19, 20). Briefly, the system consists of an inverted microscope, where a microfluidic chip with a 300 µm long constriction is assembled on a *xy*-stage. We use a channel cross-section of 20 µm x 20 µm for whole blood measurements and a 15 µm x 15 µm cross-section for lung monocytes. In all measurements a flow rate of 0.06 µl/s is applied. For each condition data from several thousand monocytes has been obtained. Only cells are analysed with an area ratio < 1.05 (ratio between actual cell area and cell area detected image analysis) to ensure a full representation of cell shape.

Derivation of elastic modulus was done using an analytical model published earlier (21). Here, the flow profile around a moving cell is calculated assuming Stokes flow and deformation is predicted from coupling the resulting stress-distribution to the cell surface applying linear elasticity theory and assuming the cell being a homogeneous material.

Data were analysed with the Software ShapeOut.

### RT-qPCR

RNA preparation was performed with Trizol (TRIzol™ Reagenz, 15596026, Invitrogen) as described before (9). The isolated RNA was reversely transcribed in cDNA using the First strand cDNA synthesis kit using M-MLV Reverse transcriptase (28025013, Invitrogen). Quantitative rtPCR analysis was performed by using SYBR™ Green PCR Master Mix (4309155, Applied Biosystems) on the system QuantStudio 7 Flex Real-Time PCR System according to the MIQE guidelines (22). Quantitative rt-PCR was performed for *Ngal* (5´-CACCACGGACTACAACCAGTTCGC-3´; 5´-TCAGTTGTCAATGCATTGGTCGGTG-3´). As house-keeping gene *Rn5s* (5´-GCCCGATCTCGTCTGATCTC-3´; 5´-GCCTATCAGCACCCGGTATTC-3´) was analysed and markers were normalized to *Rn5s*.

### Fluorescence measurement

Measurement of fluorescein conjugated concanavalin a was performed as fluorometric endpoint measurement (Ex. 485nm/Em. 520nm) in lung tissue homogenate. RFU were calculated against protein content.

### Statistical analysis

The statistical evaluation as well as the graphical presentation of the experiments were carried out in GraphPad Prism and SigmaPlot. All graphs were generated with the standard error of the mean (SE). As significance test the two-sided students T-test for unpaired samples was used, all samples p>0.05 were classified as significant. Patients’ samples were analysed by Kruskal-Wallis test followed by Dunn’s multiple comparison test, significant differences were defined by p<0.05, the correlation was tested by spearman rank order correlation. All groups were tested for normal distribution, based on the result, in parametric or non-parametric tests. Statistically significant differences for more than 3 groups were tested by one way ANOVA followed by Tukey’s multiple comparison test, or by Kruskal-Wallis test followed by Dunn’s multiple comparison test. All groups were tested against each other, all differences with a p value <0.05 were defined as statistically significant different and marked by an asterisk. Statistical analysis of RT-FDC data has been performed using linear mixed models, where we compared two groups of experimental replicates, respectively (23). While fixed effects account for the actual effect, i.e., increase or decrease in deformation, cell size or elastic modulus, random effects represent systematic or random measurement bias.

## Results

### Lung damage in a mouse model of severe acute pancreatitis

**Figure 1:**
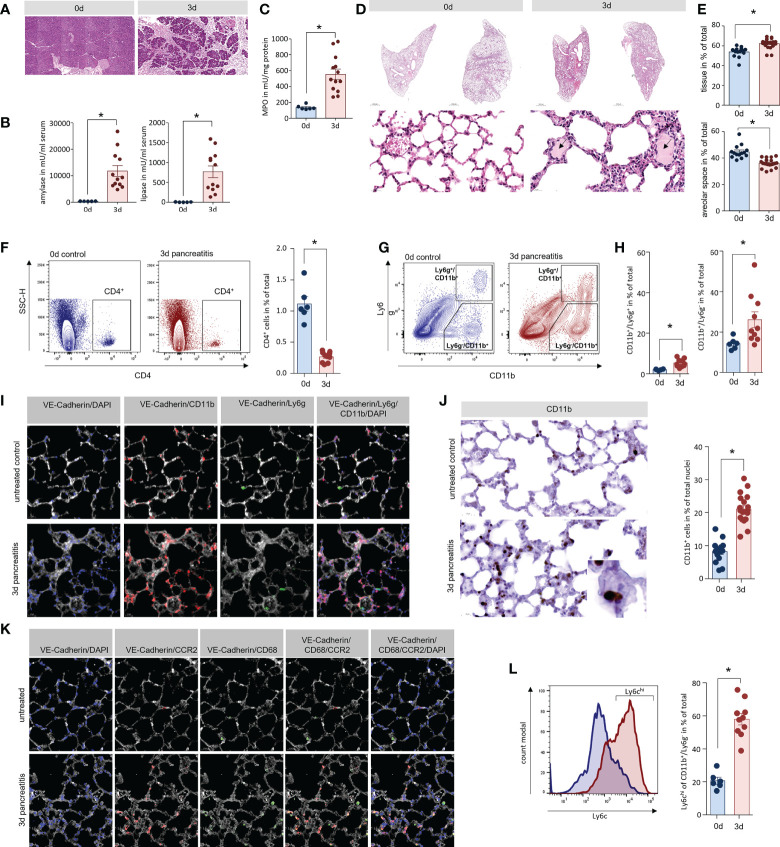
Lung damage in a mouse model of severe acute pancreatitis. **(A)** H&E staining of pancreatic sections illustrate organ damage, inflammation and acinar cell necrosis in the pancreas of mice. **(B)** Activity of amylase and lipase in serum of mice were significantly increased 3d after onset of disease. **(C)** Myeloperoxidase (MPO) activity a marker of the presence of inflammatory cells was elevated in lung tissue. **(D)** H&E staining showed a clear increase of wall thickness and oedema within the alveolus (black arrows). **(E)** An analysis of the alveolar space/tissue volume ratio showed a significant decrease of the alveolar lung volume. Classification of leukocytes from lung tissue of mice by flow cytometry analysis of surface markers. **(F)** CD4^+^ cells represent a minor population of immune cells in lung but show a significant reduction after onset of AP. **(G)** Cells of the innate immune system were distinguished by the expression of the cell surface markers Ly6g and CD11b. **(H)** The number of CD11b^+^/Ly6g ^+^ (neutrophils) as well as CD11b^+^/Ly6g^-^ cells (monocytes) are significantly increased. **(I)** Fluorescent labelling of lung tissue confirmed localisation of CD11b^+^ cells in the lung. After the induction of AP CD11b^+^/Ly6g^-^ monocytes (red) represent the majority of detectable cells in lung tissue, whereas Ly6g^+^ neutrophils (green) were observed in much lower numbers. **(J)** CD11b labelling showed a clear redistribution of CD11b^+^ cells into the interalveolar septs. Cell counts of CD11b^+^ positive cells in lungs were significantly increased after the induction of pancreatitis in mice. **(K)** After induction of AP in mice, CCR2^+^ monocytes (red) are located within the VE-Cadherin (white) positive areas of lung tissue, whereas tissue resident alveolar macrophages were labelled by CD68 (green) and are located at the border of the alveolar space. **(L)** Flow cytometry analysis of CD11b^+^/Ly6g^-^ cells in lung after induction of AP showed an increased expression of the monocyte marker Ly6c on their surface. All graphs represent 6 or more animals per group. Statistically significant differences were, tested by unpaired student`s t-test for independent samples and significance levels of p<0.05 are marked by an asterisk.

We evaluated AP-associated organ damage in an animal model of SAP with high similarity to the human situation. AP was induced by partial duct ligation with an additional hyperstimulation of caerulein (50µg/kg/bodyweight) in C57Bl/6 mice, as previously described (9–11). H&E staining of pancreas sections confirmed the development of AP 3d after the ligation of the pancreatic duct (**Figure 1A**). This was paralleled by a significant increase in serum amylase and lipase activities, which are established markers of disease severity (**Figure 1B**). Secondary organ damage was detected by a significant increase of myeloperoxidase activity in lung tissue, which is a consequence of infiltrating leukocytes (**Figure 1C**). Histologic analysis of lung sections also showed a pronounced infiltration of leukocytes 3d after the onset of pancreatitis, associated with increased alveolar wall thickness. Beside the infiltration of immune cells, oedema formation (black arrow) was a clear sign of acute lung injury (**Figure 1D**). A histological examination of the lung tissue showed a ~20% reduction of the alveolar space 3d after induction of AP (**Figure 1E**).

### Lung tissue damage is associated with infiltration of monocytes

Hyperinflammation, the very severe inflammation with cytokine storm, is believed to play a role for the induction of organ damage. To investigate the local inflammation response, we isolated leukocytes from lung tissue of AP mice and evaluated infiltrating leukocytes by flow cytometry. CD4^+^ T-cells could only be detected in negligible quantities (**Figure 1F**). The cells of the innate immune system such as neutrophils (Ly6g^+^/CD11b^+^) and monocytes/macrophages (Ly6g^-^/CD11b^+^) (**Figure 1G**). were significantly increased in lung tissue 3d after the onset of AP with CD11b^+^/Ly6g^-^ cells being the most abundant cell type (**Figure 1H**). Immunofluorescent labelling confirmed the presence of CD11b^+^ cells in lung tissue but in line with the flow cytometry data, the majority of these cells were not positive for Ly6g (**Figure 1I**). The quantification of CD11b^+^ cells by immunohistochemistry showed a significant increase of these cells within the vascular system of lung, but not within the alveolar space after onset of pancreatitis (**Figure 1J**). The C-C chemokine receptor type 2 (CCR2) is expressed on blood monocytes but not on tissue resident macrophages. Fluorescent labelling of CCR2 illustrates the accumulation of blood monocytes within the tissue, whereas CD68^+^ resident alveolar macrophages were not increased (**Figure 1K**). Ly6c, another marker of inflammatory monocytes in blood (24), showed a strong increase on Ly6c^-^/CD11b^+^ cells, underlining the presence of high numbers of blood monocytes in lung tissue after the induction of pancreatitis (**Figure 1L** **).**

### Acute kidney injury in the model of AP

**Figure 2:**
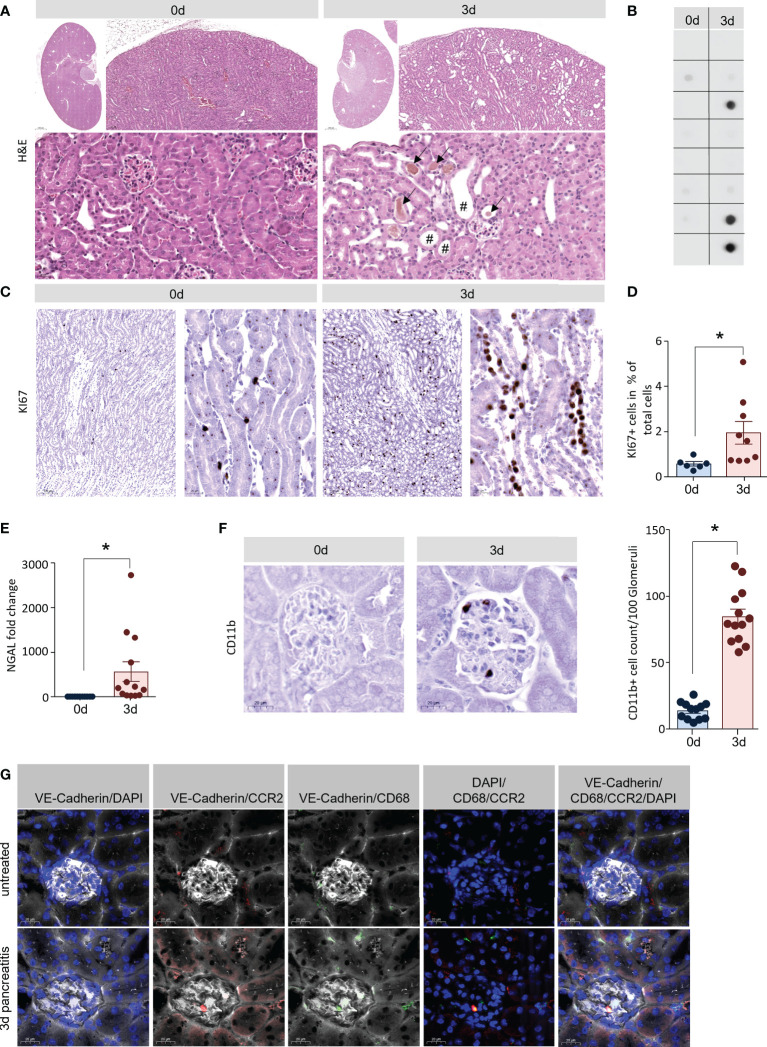
Acute kidney injury in the model of AP. **(A)** H&E staining of kidney sections showed also clear signs of early tissue damage 3d after induction of pancreatitis: tubular dilatation (rhomb) and hyaline casts (black arrows). **(B)** Dot blot analysis of cystatin C in urine samples of mice (n=8/8 for controls and SAP) were positive in 3 out of 8 mice with SAP. **(C, D)** Tubular dilatation was associated with increased cell proliferation (shown by Ki67 positive nuclei). Quantification of Ki67 staining showed a significant increase of tubular cell proliferation. **(E)** RT-qPCR analysis of *Ngal* in kidney tissue showed a significant increase 3d after onset of pancreatitis. **(F)** Kidney sections we observed a significant increase of CD11b^+^ cells in the glomerulus. **(G)** Labelling of CCR2 (red), VE-Cadherin (white) and CD68 (green) suggest that these cells are monocytes. All graphs represent 6 or more animals per group. Statistically significant differences were, tested by unpaired student`s t-test for independent samples and significance levels of p<0.05 are marked by an asterisk.

**Figure 3:**
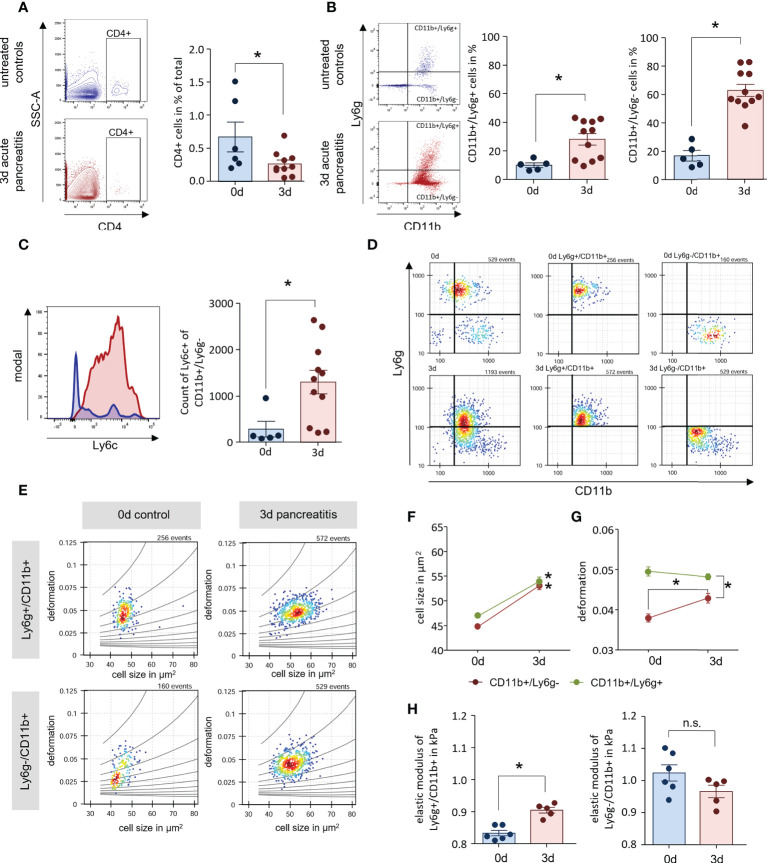
AP mice have increased numbers of enlarged blood monocytes. **(A, B)** Leukocyte detection from whole blood samples of AP mice and controls by flow cytometry analysis showed a decreased number of CD4^+^ T-cells in circulation, whereas CD11b^+^/Ly6g^+^ and CD11b^+^/Ly6g^-^ cells were found increased 3d after onset of disease. **(C)** Labelling of Ly6c within the population of CD11b^+^/Ly6g^-^, revealed a significant increase of monocytes during AP. **(D)** Their physical cell characteristics were determined by real-time fluorescence and deformability cytometry (RT-FDC). Specific Ly6g and CD11b fluorescent antibody labelling was used to discriminate neutrophils and monocytes. **(E)** Scatter plots of deformation vs. cell size for CD11b^+^/Ly6g^+^ neutrophils and CD11b^+^/Ly6g^-^ monocytes. **(F, G)** Cell size is increased during SAP in both cell types **(D)** while the increase in deformation observed in CD11b^+^/Ly6g^-^ monocytes, does not reach the deformation values of CD11b^+^/Ly6g^+^ neutrophils. **(H)** The elastic modulus is significantly increased in neutrophils but not in monocytes. All graphs represent 5 or more animals each group, significance was tested by unpaired, two tailed student`s t-test. RT-FDC data has been analysed using linear mixed models (*p<0.05). n.s. means (not significant).

H&E staining of kidney sections showed tubular dilatation (marked by rhombs) and intra-tubular deposits (marked by arrows), which are both histological signs of AKI. They were both present in tissue of mice 3d after the onset of pancreatitis, but completely absent in healthy tissue (**Figure 2A**) (25). Urinary accumulation of cystatin C, a marker of AKI (26), was detected by dot blot analysis in 3 out of 8 urine samples of pancreatitis animals, but not in control animals (**Figure 2B**). Another indicator of kidney damage was the significantly increased proliferation of tubule cells shown by Ki67 labelling (**Figures 2C**, **3D**), as well as a significantly increased gene expression of neutrophil gelatinase associated lipocalin (NGAL) (27) 3d after the onset of SAP (**Figure 2E**). To evaluate if CD11b+ cells could also be detected in kidney tissue samples like in lung we also labelled CD11b^+^ cells in tissue sections of kidney, 3d after the onset of pancreatitis, CD11b^+^ cells were significantly increased within kidney glomeruli (**Figure 2F**). Fluorescent labelling of CCR2 gave evidence that blood monocytes accumulate within the glomeruli (**Figure 2G**). In contrast to the rather mild pancreatitis model of hormonal (caerulein) hyperstimulation, the partial duct ligation in mice is associated with organ damage such as AKI and respiratory dysfunction (12), similar to the human situation.

### Accumulation of enlarged blood monocytes in AP mice

In histological sections of lung and kidney we observed an accumulation of CD11b^+^ cells within the tissue. In the following experiments we also investigated the circulating immune cells. Flow cytometric analysis of blood samples from AP mice showed similar results as for leukocytes isolated from lung tissue. CD4^+^ T-cells were significantly reduced (**Figure 3A**), whereas we could observe a slight increase of CD11b^+^/Ly6g^+^ cells and a prominent increase of CD11b^+^/Ly6g^-^ cells (**Figure 3B**). Comparable to lung tissue, the number of Ly6c^hi^ cells within the population of CD11b^+^/Ly6g^-^ cells was increased and identified these cells as monocytes (**Figure 3C**). The mechanical characteristics of myeloid derived cells have been discussed in a pathophysiological context in the recent literature (28). In further experiments we isolated blood cells from mice and analysed their mechanical properties by real-time fluorescence and deformability cytometry (RT-FDC) after gating for 1) CD11b^+^/Ly6g^+^neutrophils and 2) CD11b^+^/Ly6g^-^monocytes (**Figure 3D**) (19). Deformation and cell size were analysed in Ly6g and CD11b labelled populations indicating a general increase in cell size (**Figure 3E**). A comparison of six experimental replicates demonstrated a significant increase in cell size after 3d for all investigated populations (**Figure 3F**). Whereas CD11b^+^/Ly6g^+^ neutrophils showed a high deformation already at 0d (**Figure 3G**), the deformation of CD11b^+^/Ly6g^-^ monocytes increased after onset of pancreatitis but did not reach the level of neutrophiles. Applying an analytical model to our data enables to calculate the elastic modulus as an intrinsic material property (21). The results indicate a significant increase for enlarged CD11b^+^/Ly6g^+^ neutrophiles 3d after onset of pancreatitis, whereas the elasticity of the bigger CD11b^+^/Ly6g^-^ monocytes was not increased (**Figure 3H**).

### Enlarged monocytes accumulate in lung tissue with reduce microcirculation

**Figure 4:**
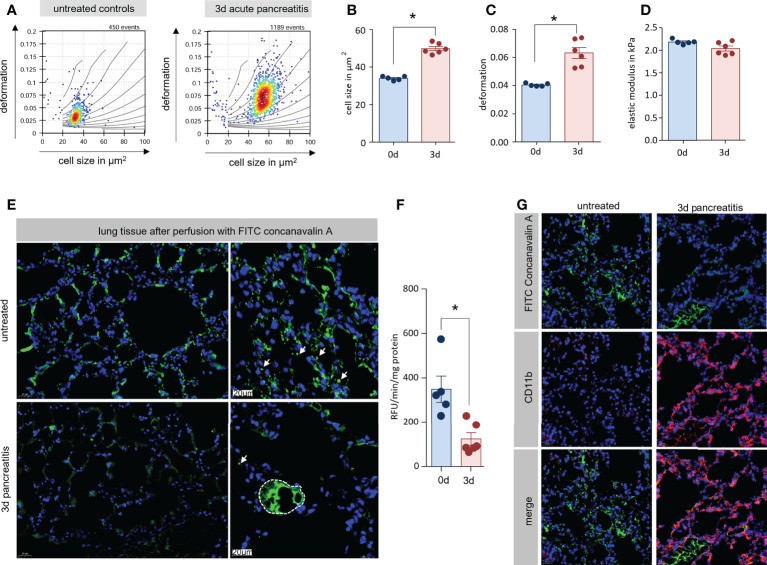
Enlarged monocytes were located in lung tissue with reduce microcirculation. **(A)** Monocytes (reflects CD11b^+^/Ly6g^-^cells) from lung tissue of mice with and without AP were isolated and analysed by RT-FDC. Deformation vs. cell size scatter plots of monocytes from lung showed the same mechanical characteristics as blood CD11b^+^/Ly6g^-^ cells (Figure 4). **(B, C)** Statistical analysis of experimental replicates indicates a significant increase in cell size and deformation. **(D)** No significant change in elastic modulus is observed. **(E)** To visualise the blood circulation in the lungs, the animals were perfused with fluorescein conjugated concanavalin a. 3d after induction of AP, only large vesicles could be stained with concanavalin a. **(F)** Quantification of fluorescence in lung tissue homogenate showed a significant reduction 3d after onset of disease. **(G)** Labelling of CD11b and FITC-concanavalin a showed a displacement of concanavalin a staining by CD11b^+^ cells. All graphs represent 5 or more animals per group, statistically significant difference was tested by unpaired, two tailed students t-test. RT-FDC data has been analysed using linear mixed models (*, p<0.05).

During pancreatitis monocytes are mobilized in high numbers from the bone marrow into the blood (**Figure 3B**). The size of these monocytes is increased (**Figure 3F**) but their flexibility is not increased accordingly (**Figure 3H**). We hypothesized that these monocytes accumulate in the lung vascular system and obstruct the microcirculation, which results in respiratory dysfunction. In the next step, we therefore isolated monocytes from lung tissue and analysed them by RT-FDC (19, 20). After the induction of AP we observed a significant increase of cell size and deformation of monocytes in lung tissue, similar to our previous observation in whole blood (**Figures 4A–C**). Importantly, we also found no significant differences in the elastic modulus (**Figure 4D**). These findings associate pancreatitis with increased monocyte size but maintained elastic modulus. As a consequence, the congestion of large and inflexible monocytes in small vessels can cause organ damage. Therefore, we wanted to investigate to which extent the blood flow in the lungs was reduced during AP. For this purpose and to make perfused capillaries visible, the animals received i.v. 200µl FITC conjugated concanavalin a. After 1 min of perfusion the lungs were removed and fixed in paraformaldehyde. Lungs from untreated animals show fluorescence in the entire lung tissue. However, this staining is significantly reduced 3d after induction of pancreatitis and especially small vessels are no longer stained (**Figure 4E**). Measurement of fluorescence in lung tissue homogenate confirms this observation (**Figure 4F**). Labelling of CD11b and FITC conjugated concanavalin an illustrated that the accumulation of CD11b+ cells in lung tissue is associated with a diminished perfusion of small vessels (**Figure 4G**).

### Leukocyte depletion ameliorates pancreatitis in mice

**Figure 5:**
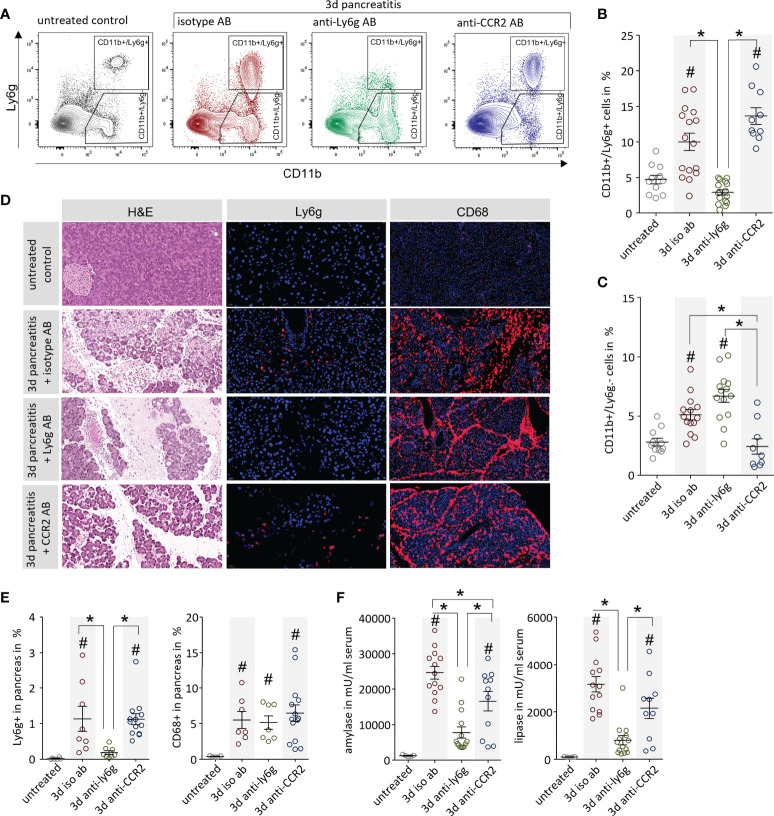
Leukocyte depletion ameliorates pancreatitis in mice. **(A)** The efficiency of the leukocyte depletion was proven by flow cytometry analysis of splenocytes with the surface markers CD11b and Ly6g to distinguish neutrophils (CD11b^+^/Ly6g^+^) and monocytes (CD11b^+^/Ly6g^-^). **(B)** An increase of CD11b^+^/Ly6g^+^ neutrophils could be observed after onset of disease in isotype and anti-CCR2 treated mice, but not after anti-Ly6g antibody treatment. **(C)** In contrast, the number of CD11b^+^/Ly6g^-^ cells was increased during pancreatitis but not in the anti-CCR2 antibody treated group. **(D)** Pancreas histology showed development of necrosis in all pancreatitis animals except in untreated controls. Immunofluorescent Ly6g-labelling showed the absence of Ly6g^+^ neutrophils in pancreatic tissue of anti-Ly6g treated mice. Labelling by anti-CD68 showed a dramatic increase of macrophages in the pancreatic tissue of all mice with pancreatitis, even after the depletion of monocytes by anti-CCR2 treatment. **(E)** Quantitative evaluation of Ly6g^+^ and CD68^+^ cells in immunofluorescence-labelled pancreatic sections **(F)** Serum amylase and lipase activity reflected disease severity and showed a reduction a dramatic in the anti-Ly6g treated mice and a slight decrease in the anti-CCR2 treated animals. All graphs represent 6 or more animals each group. A statistical significant differences was tested by one way ANOVA followed by Tukey’s multiple comparison test, or by Kruskal-Wallis test followed by Dunn’s multiple comparison test, A significance level of p<0.05 is marked by asterisk, rhombs indicate a significant difference to the untreated control.

In further experiments we analysed if a specific depletion of leukocytes can ameliorate AP and reduce organ damage. We depleted either neutrophils with an anti-Ly6g antibody (13) or monocytes *via* anti-CCR2 antibody (14) 1d before and 1d after onset of pancreatitis. Control mice were left untreated or received rat IgG2 Isotype antibody. The efficacy of the antibody-mediated depletion was confirmed by flow cytometry analysis of splenocytes (**Figure 5A**). Treatment with anti-Ly6g antibody resulted in a complete loss of only Ly6g^+^ neutrophils whereas the treatment with anti-CCR2 antibody showed a significant reduction of CD11b^+^/Ly6g^-^ cells but did not affect the population of CD11b^+^/Ly6g^+^ neutrophils (**Figures 5B, C**). Labelling of Ly6C, a specific marker of inflammatory monocytes (24), gave evidence that treatment with anti-CCR2 resulted in a complete loss of Ly6c^hi^/CD11b^+^ inflammatory monocytes in spleen (**Supplementary Figure 1**). The effect of anti-Ly6g and anti-CCR2 treatment on pancreatic damage was investigated by histological evaluation. H&E staining confirmed that the induction of pancreatic tissue damage was comparable in all animals, independent off leukocyte depletion (**Figure 5D**). Labelling of neutrophils by anti-Ly6g antibodies revealed invasion of only few neutrophils in controls, but a complete absence of neutrophilic granulocytes after depletion with anti-Ly6g antibody in pancreatic tissue (**Figure 5D**). The depletion of monocytes by anti-CCR2 antibody did not influence the number of tissue-resident CD68^+^ macrophages in the damaged pancreas (**Figure 5D**). Quantitative analysis of neutrophils/macrophages in pancreas confirmed a significant impact of the anti-Ly6g treatment on neutrophil counts but not of the anti-CCR2 treatment on pancreatic macrophages counts (**Figure 5E**). Serum amylase showed a significant reduction under both treatment conditions compared to the isotype antibody control group, whereas serum lipase was reduced in anti-Ly6g treated animals only (**Figure 5F**).

### Macrophage-dependent pro-inflammatory response

The anti-CCR2 depletion of systemic monocytes did not disrupt resident pancreatic macrophages and the local pancreatic damage therefore appeared unaffected. In fact, only a small number of pancreatic macrophages were CCR2-positive and thereby sensitive to anti-CCR2 treatment. In contrast, most CCR2-negative macrophages started to proliferate, as shown by Ki67/CD68 staining (**Supplementary Figures 2A, B**). Macrophages orchestrate the activation of the adaptive immune system (9, 11). The fact that tissue resident macrophages, but not monocyte derived macrophages, make up the majority of pancreatic macrophages also explains why anti-CCR2 treatment had no effect on T-cell activation. The expression of CD25^+^, CD69^+^ or CD25^+^/Foxp3^+^ T_reg_ cells, was not affected under treatment with anti-Ly6g or anti-CCR2 antibodies. Still, CD8α+ cytotoxic T-cells were somewhat reduced after monocyte depletion (**Supplementary Figure 2C**). Activated CD4^+^ T-cells are known to also express CCR2 (29), but we found no differences in T-cell activation of neither T_eff_ (CD69^+^) nor T_regs_ (CD25^+^/Foxp3^+^) by the treatment with anti-CCR2 antibody. Pancreatic macrophages, but not infiltrating neutrophils, trigger the T-cell response *via* their expressed cytokine profile (9, 11). Analysis of serum cytokines gave additional evidence that CCR2-depletion did not affect the systemic pro-inflammatory response (**Supplementary Figure 3**).

### Depletion of monocytes but not neutrophils ameliorate pancreatitis-induced organ damage

**Figure 6:**
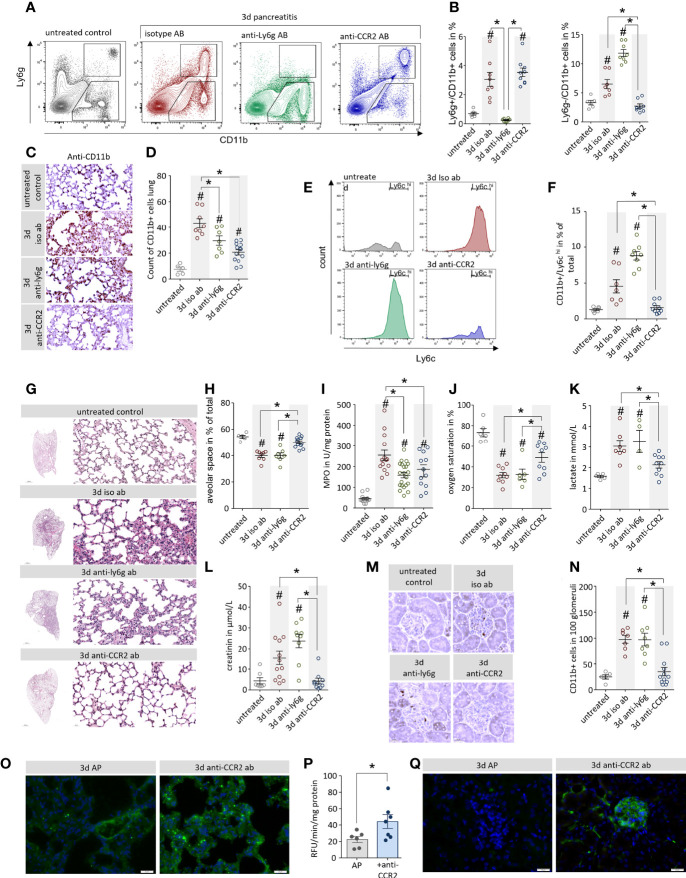
Depletion of monocytes but not neutrophils ameliorate pancreatitis-induced organ damage. **(A)** Leukocytes were isolated from lung tissue and analysed by flow cytometry using the surface markers Ly6g and CD11b. **(B)** 3d after the onset of AP a significant increase of CD11b^+^/Ly6g^+^ neutrophils could be observed in lung tissue, this increase could be blocked by the anti-Ly6g treatment. CD11b^+^/Ly6g^-^ monocytes were also increased in AP mice, this increase was blocked by anti-CCR2 treatment. **(C, D)** The specific labelling of CD11b cells confirmed the flow cytometry results and showed significant differences after quantification. **(E, F)** Flow cytometry analysis of CD11b^+^/Ly6g^-^ monocytes identify them as Ly6c^hi^ cells, a marker of inflammatory monocytes, which were completely abolished after anti-CCR2 treatment. **(G)** H&E staining of lung tissue illustrates pancreatitis induced lung damage. **(H)** The analysis of the alveolar space/tissue volume ratio showed a significant decrease of the alveolar lung volume in mice which receive isotype ab or anti-ly6g ab, but not in the anti-CCR2 ab treated group. **(I)** The pancreatitis induced increase of Myeloperoxidase activity in lung was attenuated in anti-Ly6g and anti-CCR2 treated mice. **(J)** Oxygen saturation in arterial blood samples of AP animals was reduced, but anti-CCR2 treatment improved the sO_2_ ratio compared to iso-type or anti-Ly6g treated mice. **(K)** Increased serum lactate in pancreatitis reflects a shift to anaerobic metabolism which also is ameliorated in anti-CCR2 treated mice. **(L)** Elevated serum creatinine levels in pancreatitis animals indicate kidney dysfunction which could also be ameliorated by anti-CCR2 treatment. **(M)** Labelling of CD11b in kidney sections showed reduced numbers of CD11b^+^ cells located in the glomeruli following anti-CCR2-treatment. **(N)** quantitative analysis revealed significant differences in comparison to controls. **(O)** Visualisation of the blood circulation in the lungs by perfusion with fluorescein conjugated concanavalin a showed a stronger staining of the capillary system in the ant-CCR2 treated group. **(P)** Quantification of the fluorescent signal in lung tissue homogenate showed a significant increase in the anti-CCR2 treated group 3d after onset of disease (two-tailed students t-test for independent samples). **(Q)** Fluorescein conjugated concanavalin a stained the capillary system in kidney glomeruli of anti-CCR2 treated mice but not in controls 3d after induction of AP. All graphs represent 5 or more animals each group, significance was tested by one way ANOVA followed by Tukey’s multiple comparison test, or by Kruskal-Wallis test followed by Dunn’s multiple comparison test. A significance level of p<0.05 is marked by asterisk, rhombs indicate significant difference to the untreated control mice.

In a next step we investigated the influence of neutrophil and monocyte depletion on pancreatitis-induced organ damage. We characterized leukocytes that had been isolated from lung tissue of mice by flow cytometry. Neutrophil depletion by anti-Ly6g antibody resulted in a complete loss of Ly6g^+^/CD11b^+^ neutrophils, whereas anti-CCR2 antibody treatment significantly decreased the number of Ly6c^hi^/CD11b^+^monocytes but left the population of Ly6g^+^/CD11b^+^ neutrophils unaffected (**Figures 6A, B**). Labelling of CD11b revealed a dramatic increase of CD11b+ cells in isotype and anti-Ly6g treated mice but only a minor increase in mice which received the anti-CCR2 antibody (**Figures 6C, D**). A flow cytometric characterization of the CD11b^+^/Ly6g^+^ cells by Ly6c labelling, confirmed that these cells were monocytes (24) which express high levels of Ly6c on their surface (**Figure 6E**). Whereas the depletion on neutrophils did not affect this cell-population, the treatment with anti-CCR2 resulted in a complete loss of CD11b^+^/Ly6g^-^/Ly6c^hi^ monocytes (**Figure 6F**). H&E staining of lung tissue illustrates lung damage (**Figure 6G**). Whereas isotype controls and neutrophil depleted mice showed a significant reduction of the alveolar space, which was less pronounced in anti-CCR2 antibody treated mice (**Figure 6H**). Myeloperoxidase is expressed in large amounts in monocytes and neutrophils (30) and as such a reliable marker of their tissue invasion. Measurements in lung tissue homogenate confirmed pancreatitis-induced elevated myeloperoxidase activities, which were significantly reduced in both antibody treatment groups compared to the isotype controls (**Figure 6I**). To evaluate the respiratory function/dysfunction of these mice we analysed arterial blood samples and found in all animals suffering from AP a significant reduction in oxygen saturation and increased serum lactate levels (**Figures 6J, K**). Pancreatitis animals in general had decreased pO_2_ and glucose levels, whereas pCO_2_ and ctHb were elevated (**Supplementary Figure 4A**). Serum electrolytes remained unchanged (**Supplementary Figure 4B**). While the depletion of neutrophils had no beneficial effect on respiratory function, the monocyte depletion significantly increased oxygen saturation and reduced serum lactate levels (**Figures 6J, K**). Serum creatinine levels were measured as indicator of AKI in mice. Mice which received isotype control or anti-Ly6g antibody showed a dramatic increase of serum creatinine levels, whereas creatinine levels from mice treated with anti-CCR2 antibody were comparable to untreated control mice (**Figure 6L**). Immunohistochemical labelling of CD11b identified leukocytes in kidney glomeruli of control or anti-Ly6g treated, but not of anti-CCR2 treated animals (**Figures 6M, N**). Finally, we investigated blood flow in anti-CCR2 treated mice by perfusion of the mice with 200µl FITC conjugated concanavalin a. The anti-CCR2 treated mice showed a stronger capillary staining in contrast to mice which were not depleted for monocytes (**Figure 6O**). Quantitative measurement of the fluorescence intensity in lung tissue homogenate confirms this observation (**Figure 6P**). In addition, we observed a beneficial effect of anti-CCR2 treatment also in the capillary system of kidney glomeruli (**Figure 6Q**).

### Disease severity correlates with serum monocyte attracting chemokines in patients

**Figure 7:**
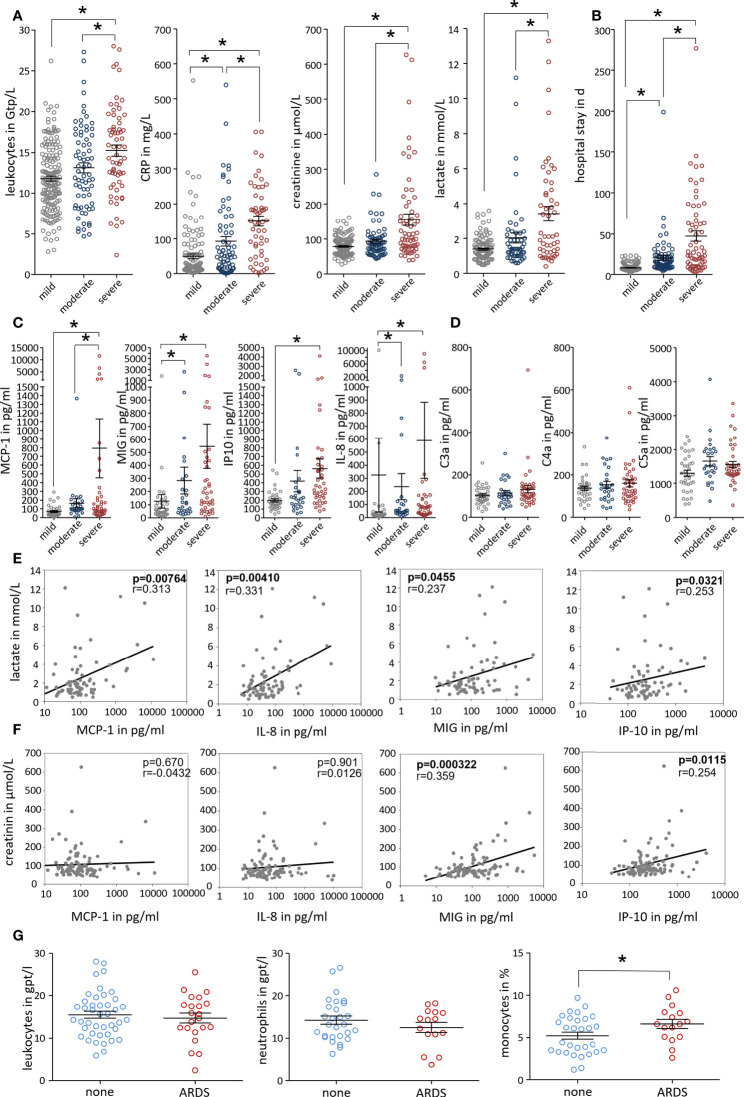
Disease severity correlates with serum monocyte attracting chemokines in patients. Patients were grouped based on the revised Atlanta classification. **(A)** Clinical parameters were evaluated from the day of admission. Inflammatory markers (leukocytes count and C-reactive protein (CRP)), serum lactate and creatinine levels were significantly increased in SAP patients. **(B)** Severe disease is also reflected by a significantly prolonged hospital stay. **(C, D)** Serum chemokines and complement cleavage products were analysed in 104 AP patients. Chemokines, MCP-1, MIG, IP-10 and IL-8 were significantly increased in SAP, whereas complement factors C3a, C4a and C5a show no severity related increase. **(E, F)** Patients samples were analysed by Kruskal-Wallis test followed by Dunn’s multiple comparison test, significant differences were defined by p<0.05 and marked by asterisk. Correlation analyses were performed for serum lactate and serum creatinine to test a possible relationship between serum chemokines and markers of organ dysfunction. A correlation was tested by spearman rank order correlation and p values are shown in each graph. **(G)** The comparison of the blood leukocyte counts in SAP patients showed that patients with ARDS have a significantly increased percentage of monocytes in the blood compared to patients without ARDS.

Finally, we investigated blood samples of 307 pancreatitis patients that had been admitted to our university hospital between 2005 and 2020 and had retrospectively been classified according to the revised Atlanta criteria into mild, moderate, and severe pancreatitis. No differences were observed between patient groups with respect to age, sex, and etiology (**Supplementary Figure 5 A**). SAP patients frequently developed AKI and respiratory failure but also cardiovascular complications were observed (**Supplementary Figure 5 B**). At the day of hospital admission leukocyte counts (Gpt/L) as well as C-reactive protein (CRP) were significantly increased in these patients. In addition, serum creatinine, indicating kidney dysfunction, and serum lactate, indicating organ hypoperfusion as a sign of critical illness, were only significantly increased in the group of patients who developed SAP (**Figure 7A**). Severe disease was also reflected by a significantly prolonged duration of hospitalisation (**Figure 7B**). We next measured the concentration of serum chemokines using Cytometric bead arrays in 104 patients. Monocyte/macrophage attracting chemokines like MCP-1 (CCL-2), MIG (CXCL-9) or IP-10 (CXCL-10) were all significantly increased in SAP patients. Neutrophil attracting chemokine IL-8 (CXCL-8) was elevated in a severity dependent manner (**Figure 7C**). Anaphylatoxins, the cleavage products of complement proteins, are also known to have chemotactic characteristics, but we found no differences for C3a, C4a and C5a with respect to disease severity (**Figure 7D**). The correlation between selected chemokines and organ dysfunction was analysed by a correlation analysis of serum lactate and creatinine. Serum lactate showed a positive correlation with MCP-1, IL-8, MIG and IP-10 (**Figure 7E**), whereas serum creatinine was significantly correlated with IP-10 and MIG (**Figure 7F**). Blood leukocytes counts were positively correlated with serum lactate and creatinine levels, whereas CRP in serum showed only a weak correlation with creatinine but none of the chemokines (**Supplementary Figures 5C–E**). From a cohort of patients with SAP, according to the criteria of the revised atlanta classification, we selected patients with respiratory organ failure (ARDS) and patients without. Patients with ARDS had an increased percentage of monocytes in the blood compared to patients without. In contrast to monocytes, the number of neutrophils or leukocytes generally was not different between the two groups (**Figure 7G**). SAP is therefore associated with a significant mobilization of leukocytes especially monocytes into the vascular system, which play an important role in the induction of secondary organ damage.

## Discussion

The induction of a multi organ dysfunction syndrome during a severe course of acute pancreatitis(4) is associated with dramatically increased morbidity and mortality and increased health care utilization(3). The underlying pathomechanism behind pancreatitis associated MODS is not well understood, a causal therapy is still missing. In our study we investigated organ damage in an AP mouse model which develops hyperinflammation (9, 11) as well as MODS, both indicating severe AP according to the revised Atlanta classification(4). AP is associated with (mainly) necrosis of acinar cells, thus leading to local tissue damage and the initiation of an overwhelming pro-inflammatory immune response. Apparently, the extent of local damage has a critical influence on the strength of the inflammatory response and is therefore suggested to play an essential role in the induction of secondary organ damage and systemic complications (2, 5, 7). It`s the overwhelming immune response of AP which defines the severity of the disease course. An activation of the transcription factor NFκB in acinar cells occurs in parallel with intra acinar protease activation, and is an early event in disease manifestations (31, 32). The release of cytokines and chemokines then recruits immune cells to the pancreas where they contribute to the local damage (9, 13, 33–35) and enhance the immune response (11, 36, 37). The activated immune response in the course of AP is similar to other severe inflammatory responses such as sepsis (38) or sever burn traumata (39) which all may comprise the induction of MODS (40, 41). This suggests a common inflammatory response mechanism leading to secondary organ dysfunction. The most affected organs are the renal and the respiratory system which have one thing in common, their vascular system of micro-capillaries. A disturbed microcirculation could contribute to organ failure (40), like we observed in lung after induction of AP in mice. Furthermore, we observed a significant increase of two cell populations of the innate immune system circulating in the blood after the onset of pancreatitis: 1.) Ly6c^hi^/CD11b^+^ monocytes which could effectively be depleted by the usage of anti-CCR2 antibody (14), and 2.) Ly6g^+^/CD11b^+^ cells which represent neutrophils and could successfully be depleted by anti-Ly6g antibody application. The pre-treatment with anti-Ly6g depleting antibody results in a complete loss of neutrophils and attenuates the local pancreatic damage but has no effect on disease related organ dysfunction. It is well known that neutrophils contribute to acinar cell damage *via* the release of reactive oxygen (33) species or pro-inflammatory cytokines like TNFα (13). Our results confirm these findings but suggest that neutrophils are not involved in the initiation of systemic complications.

In contrast, the depletion of monocytes by anti CCR2 antibody demonstrated that a macrophage-mediated immune response is much more complex. Ly6c^hi^/CD11b^+^ monocytes were depleted from spleen and lung tissue of AP mice, but the tissue-resident macrophages of the pancreas were not affected. Also, the systemic pro-inflammatory immune response did not differ between anti-CCR2 and isotype control mice, which supports the assumption that resident pancreatic macrophages initiate the development of systemic hyperinflammation by activating the adaptive immune system (11). These tissue resident macrophages (42, 43) are independent of circulating monocytes. They start to proliferate in response to acinar cell damage and orchestrate the systemic immune response. While the depletion of CCR2^+^ monocytes did neither affect the systemic immune response, nor necrosis clearance by resident pancreatic macrophages, the development of organ dysfunction was significantly reduced. Monocyte–depleted mice showed better oxygen saturation and less elevated serum creatinine levels, suggesting less pancreatitis induced lung and kidney damage. Histologically, we observed that the lung damage was also significantly lower in the monocyte-depleted animals. In particular, the alveolar space is significantly less restricted compared with the isotype controls, suggesting an improved lung function during disease. Monocytes develop from precursor cells in the bone marrow and circulate in the blood (44), whereas tissue resident macrophages develop from invading monocytes starting with embryogenesis (45). After transmigration into the tissue, they act as monocyte derived macrophages. In the adult, macrophages play a role in almost all inflammatory responses, including wound repair processes and it has been shown in adult guinea pigs that, when macrophage influx is blocked by administration of anti-macrophage serum, wound healing is severely impaired (46). Following the induction of pancreatitis we saw a tremendous release of monocytes from bone marrow into the vascular system, but, surprisingly, no rapid transmigration into the site of pancreatic damage. The depletion of these circulating monocytes did not affect the local and systemic pro-inflammatory immune response, but significantly ameliorated organ complications. These findings suggest that the induction of MODS in severe pancreatitis is not only mediated by an immunological signalling pathway but also related to the physical properties of the monocytes (28). Lung and kidney are characterised by microvascular systems and the passage of large cells, like monocytes, through these capillary systems requires a flexible adaptation to their small capillaries (47). LPS activated monocytes acquire stiffer physical properties, leading to a prolonged retention in the capillary system of the lung (48). Here we demonstrate that the induction of pancreatitis also changed the mechanical behaviour of myeloid derived cells dramatically. During pancreatitis, CD11b^+^/Ly6g^+^ neutrophils and CD11b^+^/Ly6g^-^ monocytes are all characterized by an increased cell size. However, neutrophils revealed a high deformation compared to monocytes. As a consequence, large numbers of these rather large and stiff monocytes remain circulating in the vascular system while they do not transmigrate into the pancreatic tissue. The depletion of these monocytes, but not of neutrophils significantly ameliorated lung and kidney damage. A retrospective analysis of clinical data from patients demonstrated a correlation of leukocyte count and lactate, as marker for lung damage, or serum creatinine as marker for kidney damage. In addition, the serum level of the Monocyte attracting chemokines MCP-1, which is the ligand of CCR2, was significantly increased in patients with SAP compared to milder forms of the disease. This underlines the importance of monocyte mobilisation for the disease severity and the induction of MODS in patients with SAP. Severe immune responses like in sepsis or severe trauma are also frequently associated with secondary organ damages like AKI or ARDS. We speculate that our observation is not pancreatitis specific. Monocytes are released from the bone marrow in response to the pancreatitis-induced pro-inflammation and enter the vascular system. These large numbers of monocytes, isolated from blood as well as from lung tissue, which are characterized by increased size but unchanged elasticity is especially problematic for organs with small capillary networks like the lungs or the glomeruli of kidney. We propose that these large monocytes represent a common pathomechanism and are the reason why severe immune reactions lead to damaged renal and pulmonary system as it is known for pancreatitis, burn traumata and sepsis (2, 4, 40, 49). Interestingly, Recent genome wide association studies of lung tissue from Covid-19 patients identified an association of severe disease courses with increased CCR2 expression (50). Monoclonal anti-CCR2 antibody therapy, which is already used for rheumatoid arthritis (51) may be a treatment option against MODS during severe acute pancreatitis and other inflammatory diseases.

In summary, we could demonstrate a tremendous mobilisation of monocytes from the bone marrow in response to strong pro-inflammation during AP. The number of circulating monocytes was dramatically increased after onset of pancreatitis and also their physical properties were changed. Increased cell size without a compensatory increase in elasticity results in the accumulation of monocytes in narrow capillaries of the lungs and glomeruli where they cause organ dysfunction, as characterized by AKI and ARDS in our mouse model of SAP. The depletion of monocytes, but not of neutrophils, was sufficient to significantly ameliorate lung and kidney damage. Monocytes could represent a promising therapeutic target for the prevention of organ complications in SAP and possible other inflammation-related disorders.

## Data availability statement

The original contributions presented in the study are included in the article/**Supplementary Material**. Further inquiries can be directed to the corresponding author.

## Ethics statement

The studies involving human participants were reviewed and approved by Ethics Committee of Universitymedicine Greifswald. The patients/participants provided their written informed consent to participate in this study. The animal study was reviewed and approved by LALLF- Landesamt für Landwirtschaft, Lebensmittelsicherheit und Fischerei Mecklenburg-Vorpommern.

## Author contributions

Concept of the study MS, FW, and AW. Data acquisition and interpretation: MS, AW, JG, AA, DB, OO, MN, MM, SR, BB, SFR. Writing committee MS, FW, AA, ML. Correction of manuscript and approval of final version: all. All authors contributed to the article and approved the submitted version.

## Funding

This work was supported by Deutsche Forschungsgemeinschaft (DFG SE 2702/2-1, SE 2702/2-3, AG 203/4-1 and GRK 1947, GRK 2719), the PePPP center of excellence MV (ESF/14-BM-A55-0045/16) and the EnErGie/P2 Project (ESF/14-BM-A55-0008/18), the Bundesministerium für Bildung und Forschung (ZIK HIKE grant to OO under grant agreement 03Z22CN11) and the Deutsches Zentrum für Herz-Kreislauf-Forschung (Postdoc startup grant to OO under grant agreement 81X3400107).

## Conflict of interest

OO is co-founder of Zellmechanik Dresden GmbH distributing the technology of real-time deformability cytometry.

The remaining authors declare that the research was conducted in the absence of any commercial or financial relationships that could be construed as a potential conflict of interest.

## Publisher’s note

All claims expressed in this article are solely those of the authors and do not necessarily represent those of their affiliated organizations, or those of the publisher, the editors and the reviewers. Any product that may be evaluated in this article, or claim that may be made by its manufacturer, is not guaranteed or endorsed by the publisher.

## Supplementary material

Supplement 1 To characterize the antibody treatment effect on the population of CD11b^+^/Ly6g^-^ cells we investigated the expression of the cell surface protein Ly6c on cells isolated from spleen. At 3d of pancreatitis we observed an increased expression of Ly6c on these cells which identified them as monocytes. This increase was specifically suppressed in anti-CCR2 treated animals.

Supplement 2 Pancreatic tissue macrophages were not affected by systemic anti-CCR2 monocyte depletion. **(A)** Double labelling of CD68 and CCR2 indicated that only a small percentage of resident macrophages carried CCR2 and animals treated with anti-CCR2 antibody showed a complete loss of these CCR2^+^ macrophages. **(B)** Ki67 labelling, a marker of cell proliferation, showed, that macrophages start to proliferate after the induction of pancreatitis. **(C)** Flow cytometric analysis of T-cell activation in spleen revealed a pancreatitis induced increase of CD69^+^, CD25^+^, CD25^+^/Foxp3^+^ T-helper cells and of CD8α^+^ cells in all animals, independent of a depletion of neutrophils or monocytes. All graphs represent 6 or more animals per group. Statistically significant differences were tested by one way ANOVA followed by Tukey’s multiple comparison test, or by Kruskal-Wallis test followed by Dunn’s multiple comparison test. A significance level of p<0.05 is marked by asterisk, rhombs indicate significant difference to the untreated control mice.

Supplement 3 Serum cytokines were measured by fluorometric bead array analysis in isotype treated and CCR2-treated mice. The observed increase of pro-inflammatory cytokines IL-6 and TNFα as well as of T-cell activating cytokine IL-2 during pancreatitis was not changed in isotype or CCR2-treated mice. All graphs represent 6 or more animals in each group.

Supplement 4
**(A)** Analysis of oxygen saturation, acid-alkaline balance and serum electrolytes in SAP mice. pO_2_, FO_2_Hb, glucose and pH were in general decreased after induction of pancreatitis whereas pCO_2_ and ctHb were slightly increased. **(B)** Pancreatitis seems to disturb the acid-alkaline balance, but serum electrolytes were not affected by pancreatitis, except for Na^+^ which showed a slight increase. All graphs represent 6 or more animals each group. Differences were tested by one way ANOVA followed by Tukey’s multiple comparison test, or by Kruskal-Wallis test followed by Dunn’s multiple comparison test, A significance level of p<0.05 is marked by asterisk, rhombs indicate significant difference to the untreated control mice.

Supplement 5 Patients were grouped based on the revised Atlanta classification. **A** The patient groups (mild moderate and severe) did not differ in terms of composition (age, gender or etiology). **(B)** Acute kidney injury (AKI), acuterespiratory distress syndrome (ARDS), systemic inflammatory response syndrome (SIRS) and Sepsis were common complications in the group of severe acute pancreatitis patients. Blood and serum markers of pancreatitis patients were recorded at day of hospital admission. **(C)** Spearman rank order correlation tests confirmed a positive correlation for serum lactate and serum creatinine to total blood leukocyte count. **(D)** C-reactive protein (CRP), a classical acute phase protein, also correlated with creatinine, but not with serum lactate. **(E)** All tested chemokines (MCP-1, MIG, IP-10 and IL-8) did not correlate with the CRP level, which indicates that the leukocyte mobilisation occurs independent from the acute phase protein. p values are located in the upper right corner of each graph.

## References

[1] van Dijk SM Hallensleben NDL van Santvoort HC Fockens P van Goor H Bruno MJ. Acute pancreatitis: recent advances through randomised trials. Gut (2017) 66:2024–32. doi: 10.1136/gutjnl-2016-313595 28838972

[2] Garg PK Singh VP. Organ failure due to systemic injury in acute pancreatitis. Gastroenterology (2019) 156:2008–23. doi: 10.1053/j.gastro.2018.12.041 PMC6486861 30768987

[3] Peery AF Crockett SD Murphy CC Lund JL Dellon ES Williams JL. Burden and cost of gastrointestinal, liver, and pancreatic diseases in the united states: Update 2018. Gastroenterology (2019) 156:254–272.e11. doi: 10.1053/j.gastro.2018.08.063 30315778 PMC6689327

[4] Banks PA Bollen TL Dervenis C Gooszen HG Johnson CD Sarr MG. Classification of acute pancreatitis–2012: revision of the Atlanta classification and definitions by international consensus. Gut (2013) 62:102–11. doi: 10.1136/gutjnl-2012-302779 23100216

[5] Bhatia M Moochhala S. Role of inflammatory mediators in the pathophysiology of acute respiratory distress syndrome. J Pathol (2004) 202:145–56. doi: 10.1002/path.1491 14743496

[6] Schepers NJ Bakker OJ Besselink MG Ahmed Ali U Bollen TL Gooszen HG. Impact of characteristics of organ failure and infected necrosis on mortality in necrotising pancreatitis. Gut (2019) 68:1044–51. doi: 10.1136/gutjnl-2017-314657 29950344

[7] Zhang H Neuhöfer P Song L Rabe B Lesina M Kurkowski MU. IL-6 trans-signaling promotes pancreatitis-associated lung injury and lethality. J Clin Invest (2013) 123:1019–31. doi: 10.1172/JCI64931 PMC3582130 23426178

[8] Frossard JL Lenglet S Montecucco F Steffens S Galan K Pelli G. Role of CCL-2, CCR-2 and CCR-4 in cerulein-induced acute pancreatitis and pancreatitis-associated lung injury. J Clin Pathol (2011) 64:387–93. doi: 10.1136/jcp.2010.088500 21345872

[9] Sendler M Weiss F-U Golchert J Homuth G van den Brandt C Mahajan UM. Cathepsin b-mediated activation of trypsinogen in endocytosing macrophages increases severity of pancreatitis in mice. Gastroenterology (2018) 154:704–718.e10. doi: 10.1053/j.gastro.2017.10.018 29079517 PMC6663074

[10] Sendler M Beyer G Mahajan UM Kauschke V Maertin S Schurmann C. Complement component 5 mediates development of fibrosis, *via* activation of stellate cells, in 2 mouse models of chronic pancreatitis. Gastroenterology (2015) 149:765–776.e10. doi: 10.1053/j.gastro.2015.05.012 26001927 PMC4560830

[11] Sendler M van den Brandt C Glaubitz J Wilden A Golchert J Weiss FU. NLRP3 inflammasome regulates development of systemic inflammatory response and compensatory anti-inflammatory response syndromes in mice with acute pancreatitis. Gastroenterology (2020) 158:253–69.e14. doi: 10.1053/j.gastro.2019.09.040 31593700

[12] Glaubitz J Wilden A van den Brandt C Weiss FU Bröker BM Mayerle J. Experimental pancreatitis is characterized by rapid T cell activation, Th2 differentiation that parallels disease severity, and improvement after CD4+ T cell depletion. Pancreatol Off J Int Assoc Pancreatol IAP Al (2020) 20(8):1637–47. doi: 10.1016/j.pan.2020.10.044 33097430

[13] Sendler M Dummer A Weiss FU Krüger B Wartmann T Scharffetter-Kochanek K. Tumour necrosis factor α secretion induces protease activation and acinar cell necrosis in acute experimental pancreatitis in mice. Gut (2013) 62:430–9. doi: 10.1136/gutjnl-2011-300771 22490516

[14] Mack M Cihak J Simonis C Luckow B Proudfoot AE Plachý J. Expression and characterization of the chemokine receptors CCR2 and CCR5 in mice. J Immunol Baltim Md 1950 (2001) 166:4697–704. doi: 10.4049/jimmunol.166.7.4697 11254730

[15] Glaubitz J Wilden A Golchert J Homuth G Völker U Bröker BM. In mouse chronic pancreatitis CD25+FOXP3+ regulatory T cells control pancreatic fibrosis by suppression of the type 2 immune response. Nat Commun (2022) 13:4502. doi: 10.1038/s41467-022-32195-2 35922425 PMC9349313

[16] Cossarizza A Chang H-D Radbruch A Abrignani S Addo R Akdis M. Guidelines for the use of flow cytometry and cell sorting in immunological studies (third edition). Eur J Immunol (2021) 51:2708–3145. doi: 10.1002/eji.202170126 34910301 PMC11115438

[17] Aghdassi AA John DS Sendler M Weiss FU Reinheckel T Mayerle J. Cathepsin d regulates cathepsin b activation and disease severity predominantly in inflammatory cells during experimental pancreatitis. J Biol Chem (2018) 293:1018–29. doi: 10.1074/jbc.M117.814772 PMC5777244 29229780

[18] Sendler M Maertin S John D Persike M Weiss FU Krüger B. Cathepsin b activity initiates apoptosis *via* digestive protease activation in pancreatic acinar cells and experimental pancreatitis. J Biol Chem (2016) 291:14717–31. doi: 10.1074/jbc.M116.718999 PMC4938190 27226576

[19] Rosendahl P Plak K Jacobi A Kraeter M Toepfner N Otto O. Real-time fluorescence and deformability cytometry. Nat Methods (2018) 15:355–8. doi: 10.1038/nmeth.4639 29608556

[20] Otto O Rosendahl P Mietke A Golfier S Herold C Klaue D. Real-time deformability cytometry: on-the-fly cell mechanical phenotyping. Nat Methods (2015) 12:199–202. doi: 10.1038/nmeth.3281 25643151

[21] Mietke A Otto O Girardo S Rosendahl P Taubenberger A Golfier S. Extracting cell stiffness from real-time deformability cytometry: Theory and experiment. Biophys J (2015) 109:2023–36. doi: 10.1016/j.bpj.2015.09.006 PMC4656812 26588562

[22] Bustin SA Benes V Garson JA Hellemans J Huggett J Kubista M. The MIQE guidelines: minimum information for publication of quantitative real-time PCR experiments. Clin Chem (2009) 55:611–22. doi: 10.1373/clinchem.2008.112797 19246619

[23] Herbig M Mietke A Müller P Otto O. Statistics for real-time deformability cytometry: Clustering, dimensionality reduction, and significance testing. Biomicrofluidics (2018) 12:042214. doi: 10.1063/1.5027197 29937952 PMC5999349

[24] Geissmann F Jung S Littman DR. Blood monocytes consist of two principal subsets with distinct migratory properties. Immunity (2003) 19:71–82. doi: 10.1016/s1074-7613(03)00174-2 12871640

[25] Moeckel GW. Pathologic perspectives on acute tubular injury assessment in the kidney biopsy. Semin Nephrol (2018) 38:21–30. doi: 10.1016/j.semnephrol.2017.09.003 29291758

[26] Koyner JL Bennett MR Worcester EM Ma Q Raman J Jeevanandam V. Urinary cystatin c as an early biomarker of acute kidney injury following adult cardiothoracic surgery. Kidney Int (2008) 74:1059–69. doi: 10.1038/ki.2008.341 PMC2745082 18650797

[27] Nguyen MT Devarajan P. Biomarkers for the early detection of acute kidney injury. Pediatr Nephrol (2008) 23:2151–7. doi: 10.1007/s00467-007-0470-x PMC6904376 17394022

[28] Bashant KR Toepfner N Day CJ Mehta NN Kaplan MJ Summers C. The mechanics of myeloid cells. Biol Cell (2020) 112:103–12. doi: 10.1111/boc.201900084 31916263

[29] Bakos E Thaiss CA Kramer MP Cohen S Radomir L Orr I. CCR2 regulates the immune response by modulating the interconversion and function of effector and regulatory T cells. J Immunol Baltim Md 1950 (2017) 198:4659–71. doi: 10.4049/jimmunol.1601458 28507030

[30] Sugiyama S Okada Y Sukhova GK Virmani R Heinecke JW Libby P. Macrophage myeloperoxidase regulation by granulocyte macrophage colony-stimulating factor in human atherosclerosis and implications in acute coronary syndromes. Am J Pathol (2001) 158:879–91. doi: 10.1016/S0002-9440(10)64036-9 PMC1850342 11238037

[31] Gukovsky I Gukovskaya AS Blinman TA Zaninovic V Pandol SJ. Early NF-kappaB activation is associated with hormone-induced pancreatitis. Am J Physiol (1998) 275:G1402–1414. doi: 10.1152/ajpgi.1998.275.6.G1402 9843778

[32] Steinle AU Weidenbach H Wagner M Adler G Schmid RM. NF-kappaB/Rel activation in cerulein pancreatitis. Gastroenterology (1999) 116:420–30. doi: 10.1016/S0016-5085(99)70140-X 9922324

[33] Gukovskaya AS Vaquero E Zaninovic V Gorelick FS Lusis AJ Brennan M-L. Neutrophils and NADPH oxidase mediate intrapancreatic trypsin activation in murine experimental acute pancreatitis. Gastroenterology (2002) 122:974–84. doi: 10.1053/gast.2002.32409 11910350

[34] Wu J Zhang L Shi J He R Yang W Habtezion A. Macrophage phenotypic switch orchestrates the inflammation and repair/regeneration following acute pancreatitis injury. EBioMedicine (2020) 58:102920. doi: 10.1016/j.ebiom.2020.102920 32739869 PMC7399125

[35] John DS Aschenbach J Krüger B Sendler M Weiss FU Mayerle J. Deficiency of cathepsin c ameliorates severity of acute pancreatitis by reduction of neutrophil elastase activation and cleavage of e-cadherin. J Biol Chem (2019) 294:697–707. doi: 10.1074/jbc.RA118.004376 30455353 PMC6333881

[36] Barreto SG Habtezion A Gukovskaya A Lugea A Jeon C Yadav D. Critical thresholds: key to unlocking the door to the prevention and specific treatments for acute pancreatitis. Gut (2021) 70:194–203. doi: 10.1136/gutjnl-2020-322163 32973069 PMC7816970

[37] Mayerle J Sendler M Hegyi E Beyer G Lerch MM Sahin-Tóth M. Genetics, cell biology, and pathophysiology of pancreatitis. Gastroenterology (2019) 156:1951–68.e1. doi: 10.1053/j.gastro.2018.11.081 30660731 PMC6903413

[38] Hotchkiss RS Monneret G Payen D. Sepsis-induced immunosuppression: from cellular dysfunctions to immunotherapy. Nat Rev Immunol (2013) 13:862–74. doi: 10.1038/nri3552 PMC4077177 24232462

[39] Xiao W Mindrinos MN Seok J Cuschieri J Cuenca AG Gao H. A genomic storm in critically injured humans. J Exp Med (2011) 208:2581–90. doi: 10.1084/jem.20111354 PMC3244029 22110166

[40] Rossaint J Zarbock A. Pathogenesis of multiple organ failure in sepsis. Crit Rev Immunol (2015) 35:277–91. doi: 10.1615/critrevimmunol.2015015461 26757392

[41] Wang H Ma S. The cytokine storm and factors determining the sequence and severity of organ dysfunction in multiple organ dysfunction syndrome. Am J Emerg Med (2008) 26:711–5. doi: 10.1016/j.ajem.2007.10.031 18606328

[42] Zhu Y Herndon JM Sojka DK Kim K-W Knolhoff BL Zuo C. Tissue-resident macrophages in pancreatic ductal adenocarcinoma originate from embryonic hematopoiesis and promote tumor progression. Immunity (2017) 47:323–338.e6. doi: 10.1016/j.immuni.2017.07.014 28813661 PMC5578409

[43] Davies LC Jenkins SJ Allen JE Taylor PR. Tissue-resident macrophages. Nat Immunol (2013) 14:986–95. doi: 10.1038/ni.2705 PMC4045180 24048120

[44] Jakubzick CV Randolph GJ Henson PM. Monocyte differentiation and antigen-presenting functions. Nat Rev Immunol (2017) 17:349–62. doi: 10.1038/nri.2017.28 28436425

[45] Hopkinson-Woolley J Hughes D Gordon S Martin P. Macrophage recruitment during limb development and wound healing in the embryonic and foetal mouse. J Cell Sci (1994) 107(Pt 5):1159–67. doi: 10.1242/jcs.107.5.1159 7929625

[46] Leibovich SJ Ross R. The role of the macrophage in wound repair. a study with hydrocortisone and antimacrophage serum. Am J Pathol (1975) 78:71–100. 1109560 PMC1915032

[47] Dupire J Puech P-H Helfer E Viallat A. Mechanical adaptation of monocytes in model lung capillary networks. Proc Natl Acad Sci U.S.A. (2020) 117:14798–804. doi: 10.1073/pnas.1919984117 PMC7334492 32554496

[48] Doherty DE Downey GP Schwab B Elson E Worthen GS. Lipolysaccharide-induced monocyte retention in the lung. role of monocyte stiffness, actin assembly, and CD18-dependent adherence. J Immunol Baltim Md 1950 (1994) 153:241–55. 7911494

[49] Sapan HB Paturusi I Jusuf I Patellongi I Massi MN Pusponegoro AD. Pattern of cytokine (IL-6 and IL-10) level as inflammation and anti-inflammation mediator of multiple organ dysfunction syndrome (MODS) in polytrauma. Int J Burns Trauma (2016) 6:37–43. 27335696 PMC4913232

[50] Pairo-Castineira E Clohisey S Klaric L Bretherick AD Rawlik K Pasko D. Genetic mechanisms of critical illness in COVID-19. Nature (2021) 591:92–8. doi: 10.1038/s41586-020-03065-y 33307546

[51] Vergunst CE Gerlag DM Lopatinskaya L Klareskog L Smith MD van den Bosch F. Modulation of CCR2 in rheumatoid arthritis: a double-blind, randomized, placebo-controlled clinical trial. Arthritis Rheum (2008) 58:1931–9. doi: 10.1002/art.23591 18576354

