# Supplementary figures and images for: Mobilization of CD11b+/Ly6chi monocytes causes multi organ dysfunction syndrome in acute pancreatitis
Source: Front Immunol. 2022 Oct 10;13:991295. doi: 10.3389/fimmu.2022.991295 (PMC9589437; doi:10.3389/fimmu.2022.991295)

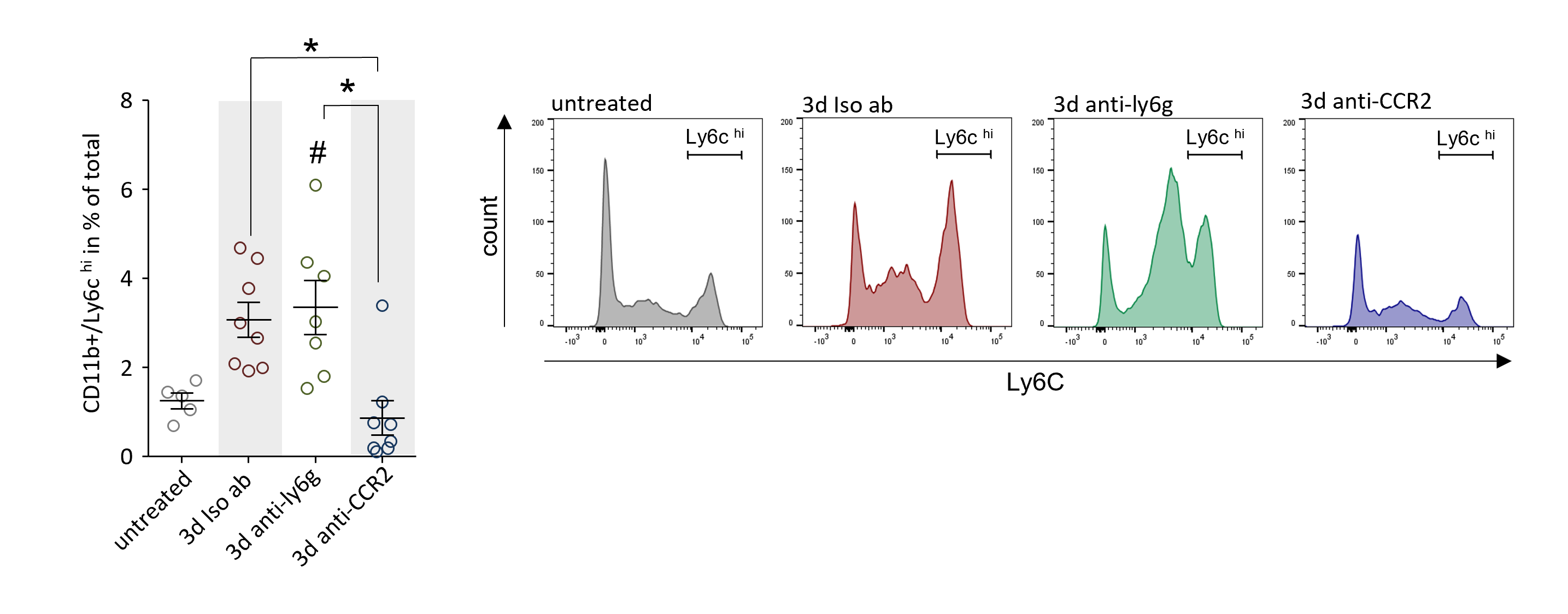

Supplement: Supplement 1 — To characterize the antibody treatment effect on the population of CD11b+/Ly6g- cells we investigated the expression of the cell surface protein Ly6c on cells isolated from spleen. At 3d of pancreatitis we observed an increased expression of Ly6c on these cells which identified them as monocytes. This increase was specifically suppressed in anti-CCR2 treated animals. [file Image_1.tif]

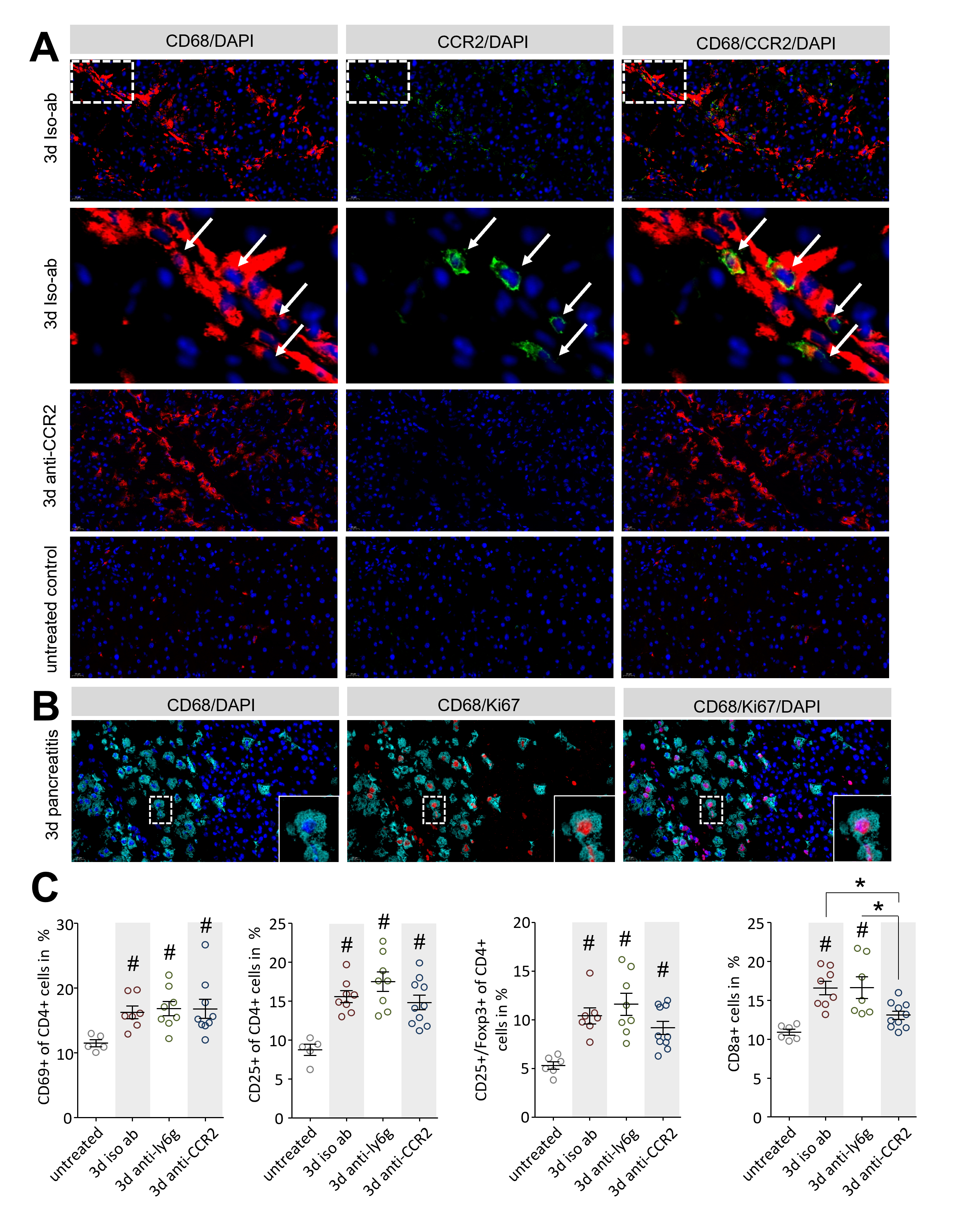

Supplement: Supplement 2 — Pancreatic tissue macrophages were not affected by systemic anti-CCR2 monocyte depletion. (A) Double labelling of CD68 and CCR2 indicated that only a small percentage of resident macrophages carried CCR2 and animals treated with anti-CCR2 antibody showed a complete loss of these CCR2+ macrophages. (B) Ki67 labelling, a marker of cell proliferation, showed, that macrophages start to proliferate after the induction of pancreatitis. (C) Flow cytometric analysis of T-cell activation in spleen revealed a pancreatitis induced increase of CD69+, CD25+, CD25+/Foxp3+ T-helper cells and of CD8α+ cells in all animals, independent of a depletion of neutrophils or monocytes. All graphs represent 6 or more animals per group. Statistically significant differences were tested by one way ANOVA followed by Tukey’s multiple comparison test, or by Kruskal-Wallis test followed by Dunn’s multiple comparison test. A significance level of p<0.05 is marked by asterisk, rhombs indicate significant difference to the untreated control mice. [file Image_2.tif]

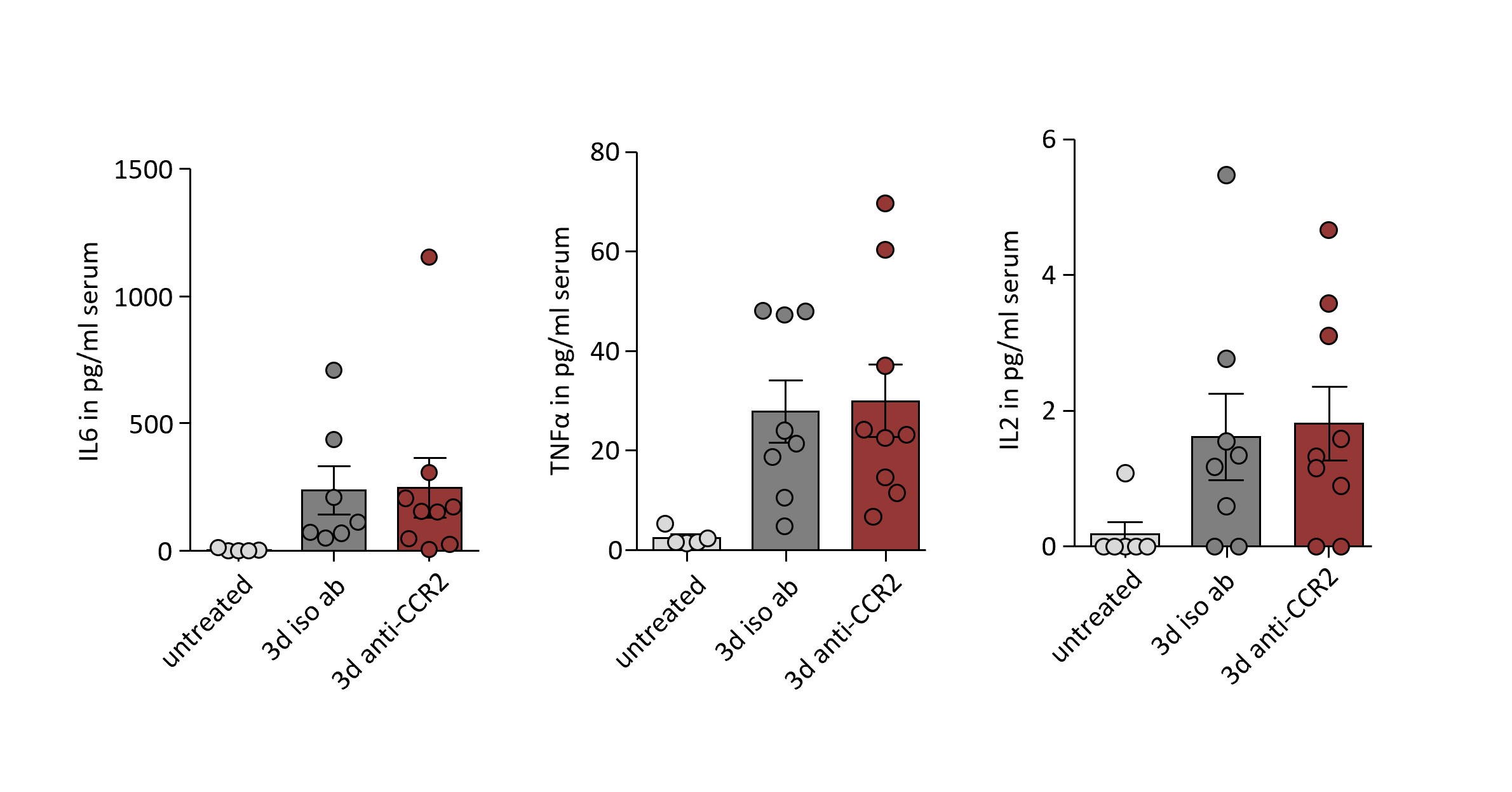

Supplement: Supplement 3 — Serum cytokines were measured by fluorometric bead array analysis in isotype treated and CCR2-treated mice. The observed increase of pro-inflammatory cytokines IL-6 and TNFα as well as of T-cell activating cytokine IL-2 during pancreatitis was not changed in isotype or CCR2-treated mice. All graphs represent 6 or more animals in each group. [file Image_3.tif]

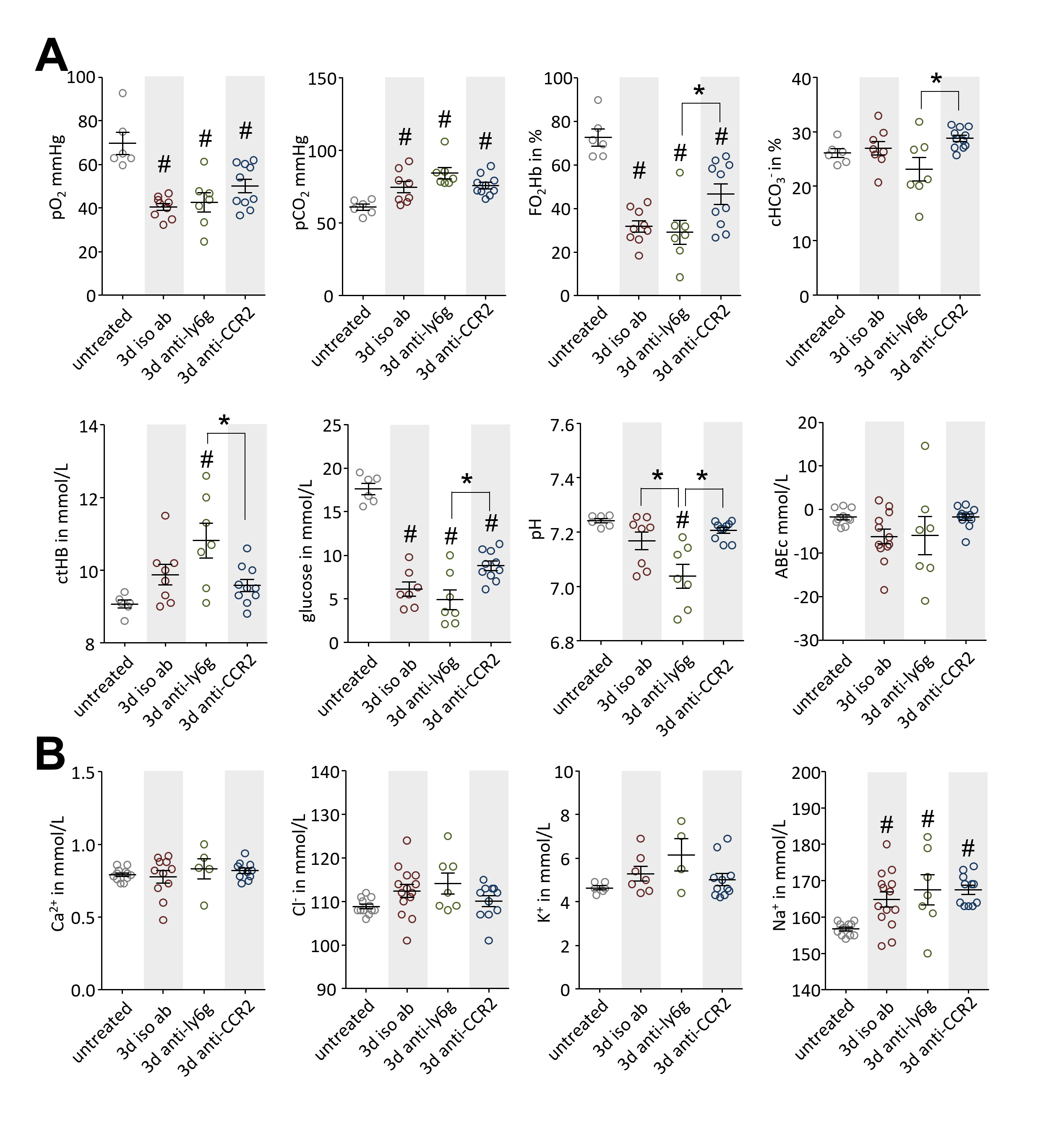

Supplement: Supplement 4 — (A) Analysis of oxygen saturation, acid-alkaline balance and serum electrolytes in SAP mice. pO2, FO2Hb, glucose and pH were in general decreased after induction of pancreatitis whereas pCO2 and ctHb were slightly increased. (B) Pancreatitis seems to disturb the acid-alkaline balance, but serum electrolytes were not affected by pancreatitis, except for Na+ which showed a slight increase. All graphs represent 6 or more animals each group. Differences were tested by one way ANOVA followed by Tukey’s multiple comparison test, or by Kruskal-Wallis test followed by Dunn’s multiple comparison test, A significance level of p<0.05 is marked by asterisk, rhombs indicate significant difference to the untreated control mice. [file Image_4.tif]

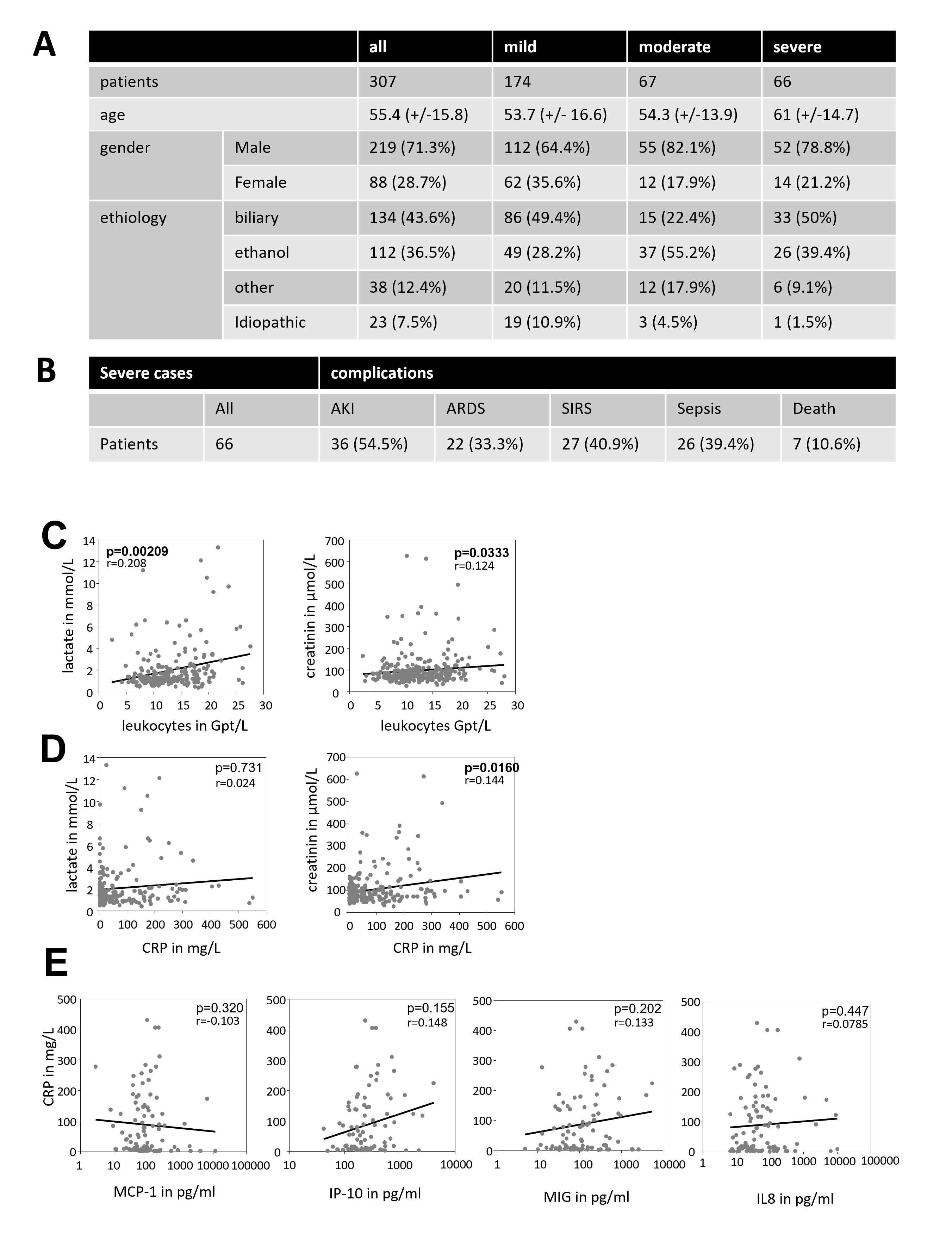

Supplement: Supplement 5 — Patients were grouped based on the revised Atlanta classification. A The patient groups (mild moderate and severe) did not differ in terms of composition (age, gender or etiology). (B) Acute kidney injury (AKI), acuterespiratory distress syndrome (ARDS), systemic inflammatory response syndrome (SIRS) and Sepsis were common complications in the group of severe acute pancreatitis patients. Blood and serum markers of pancreatitis patients were recorded at day of hospital admission. (C) Spearman rank order correlation tests confirmed a positive correlation for serum lactate and serum creatinine to total blood leukocyte count. (D) C-reactive protein (CRP), a classical acute phase protein, also correlated with creatinine, but not with serum lactate. (E) All tested chemokines (MCP-1, MIG, IP-10 and IL-8) did not correlate with the CRP level, which indicates that the leukocyte mobilisation occurs independent from the acute phase protein. p values are located in the upper right corner of each graph. [file Image_5.tif]
